# Assessment of antigen immunogenicity formulated in minigenes transfected into antigen-presenting cells

**DOI:** 10.1371/journal.pone.0321392

**Published:** 2025-04-07

**Authors:** María A. Villota-Alava, María A. Alfaro-Marenco, Carlos A. Clavijo-Ramírez, Manuel A. Patarroyo, Carlos A. Parra-López

**Affiliations:** 1 Department of Microbiology, Universidad Nacional de Colombia, School of Medicine, Immunology and Translational Medicine Research Group, Bogotá, Colombia; 2 Department of Biology, Faculty of Science, Universidad Nacional de Colombia, Bogotá, Colombia; 3 Department of Microbiology, Faculty of Medicine, Universidad Nacional de Colombia, Bogotá, Colombia; 4 Grupo de Investigación Básica en Biología Molecular e Inmunología (GIBBMI), Fundación Instituto de Inmunología de Colombia (FIDIC), Bogotá, Colombia; 5 Health Sciences Faculty, Universidad de Ciencias Aplicadas y Ambientales (U.D.C.A), Bogotá, Colombia; Pennsylvania State University Hershey Medical Center, UNITED STATES OF AMERICA

## Abstract

Tumor cells exhibit deficient antigen presentation to T cells, significantly contributing to immune evasion and tumor genesis. Peptide pulsed Antigen-presenting cells (APCs) are commonly used in cancer immunotherapy to circumvent the defects of tumor cells in processing and presenting antigens to T lymphocytes. However, peptides do not always represent epitopes naturally processed by tumor cells, which might reduce the identification of actual immunogenic antigens. Minigenes encoding concatenated immunogenic tumor epitope sequences offer a promising alternative to select tumor antigens naturally processed and presented to T cells. Hence, using APCs transfected with minigenes might contribute to immunotherapy’s effectiveness, avoiding non-naturally processed epitopes as vaccine candidates. This study evaluates APCs transfected with a minigene construct encoding HLA-A0201-restricted immunogenic antigens to stimulate antigen-specific CD8+ T lymphocytes *in vitro*. Artificial APCs (aAPCs) were also designed by co-transfecting the HEK293 cell line with plasmids encoding co-stimulatory molecules (CD80, CD83, CD137L) to assess CD8+ T cell activation efficiency, intracellular cytokine production, cytotoxic activity, activation and exhaustion marker expression. In this study, we successfully implemented a transfection methodology of HEK293 cells with a minigene encoding viral and tumor HLA-A * 0201 epitopes. These cells, used as aAPCs, allow studying the expansion and the phenotype of antigen-specific CD8+ T cells. However, our results indicate that epitope presentation alone is sufficient to activate CD8+ T cells, suggesting that the presence of co-stimulatory molecules may not be necessary for effective T cell activation. Considering that the use of HEK293 cells as aAPCs has yet to be explored and due to their high transfection efficiency with minigenes, the methodology implemented in this work enables their use to identify naturally processed immunogenic neoantigens. We believe our findings can contribute to selecting and designing personalized vaccines based on tumor neoantigens that are useful for cancer immunotherapy.

## Introduction

Immunotherapy has emerged as a promising strategy in cancer treatment, distinguished by its ability to enhance the immune response against tumors. The limitations of traditional approaches such as surgery, radiotherapy, and chemotherapy have led immunotherapy to become a new cornerstone in cancer treatment, increasing patients’ hope and quality of life [1]. The primary goal of immunotherapy is to enhance or restore the immune system’s ability to detect and destroy cancer cells, overcoming the evasion mechanisms developed by these cells to suppress the immune response [2]. The focus of this new pillar of cancer treatment has been to increase the frequency of tumor antigen-specific T lymphocytes through the administration of therapeutic vaccines based on dendritic cells, adjuvant treatment with cytokines such as IL-2, and adoptive transfer of Tumor-Infiltrating Lymphocytes (TILs) [3].

Particularly concerning therapeutic vaccines, their primary objective is to improve the quality of tumor-associated or tumor-specific antigen presentation to T cells to activate them and stimulate an anti-tumor responses [4]. Therapeutic vaccines based on dendritic cells (DCs) or other antigen-presenting cells (APCs) serve as antigen delivery systems stimulating anti-tumor T cells [5]. These cells present antigens in the major histocompatibility complex (MHC) context and act as adjuvants, releasing cytokines and chemokines to stimulate more comprehensive T-cell responses [6].

While trials using DCs in cancer vaccines have shown limited therapeutic utility, recent results of DCs pulsed with tumor neoantigens as a vaccine in the melanoma model may represent a new opportunity for DCs in cancer immunotherapy [7,8]. However, high production costs, the short lifespan of DCs, their variability among patients, storage and transportation difficulties, and issues related to antigen delivery to DCs continue to affect the implementation of DC-based vaccines [9]. Regarding antigen formulation, because of the relatively simple synthesis and low cost, DCs pulsed with synthetic peptides representing tumor antigens are often used for identifying T lymphocytes that recognize them both *ex vivo* and *in vitro*. However, synthetic peptides as antigens bypass the processing and presentation in the context of the MHC molecules exerted naturally by tumor cells or professional APCs that cross-present tumor antigens in vivo, which might lead to T cells that recognize synthetic peptides not necessarily generated *in vivo* [10]. This last limitation may bias the selection of immunogenic neoantigens for use in synthetic peptide format as vaccines and the reason why the identification of neoantigen-specific T cells in vivo in patients vaccinated with peptides reaches only 6% of those used as a vaccine [11]. This suggests that most of the tumor neoantigens predicted by bioinformatics tools are not processed or presented in MHC molecules naturally.

In this context, minigenes made of DNA sequences encoding minimal cytotoxic T lymphocyte (CTL) epitopes organized in tandem have emerged as an alternative to overcome the limitations of using peptides as antigens. Unlike complete proteins or synthetic peptides, minigenes allow the inclusion of multiple immunogenic epitopes with high affinity and good binding stability to MHC molecules, whose presentation depends on their natural processing by transfected APCs. [12].

Moreover, minigenes offer the possibility of evaluating a more significant number of candidates for immunotherapy and the advantage of being able to produce multiple copies of the antigen processed naturally [13]. Additionally, minigene-activated APCs can induce cytokine production, stimulating intracellular pattern recognition receptors (PRRs) [14] and favor the expression of co-stimulatory molecules, which are essential for inducing a robust anti-tumor T-cell response. In this context, this study aimed to construct a minigene encoding tumor-associated and viral antigens, co-transfected with plasmids encoding co-stimulatory molecules, to evaluate the ability of the transfected cells, used as aAPCs, to effectively stimulate antigen-specific T lymphocytes [15].

Overall, this study allowed us to examine the performance of aAPCs transfected with a multi-epitope minigene as an antigen delivery tool to APCs and the monitoring of LT-CD8+ specific to naturally processed antigens.

## Materials and methods

### Ethics statement

This research project is conducted in accordance with Resolution No. 008430 of 1993, which establishes the *Scientific, Technical, and Administrative Standards for Health Research* in Colombia. Chapter I addresses the ethical aspects of research involving human subjects. Based on Article 11b, this study is classified as Minimal Risk Research, as the samples used in this study were derived from Buffy coat units from healthy donors who voluntarily donated blood during campaigns organized by the District Institute of Science, Biotechnology, and Innovation in Health (IDCBIS) in Bogotá.

IDCBIS regularly conducts blood donation campaigns at various locations throughout Bogotá. These blood samples are collected in blood bags containing anticoagulants and transported to the district blood center. Once collected, the blood undergoes a fractionation process, where the components necessary for patient transfusion are separated and stored. The fraction is not used for transfusion, in this case, the Buffy coat, is typically discarded.

Since all blood donors at IDCBIS sign an informed consent form at the time of donation, explicitly authorizing the use of their blood and its derivatives for research purposes, it was not necessary to obtain additional informed consent from the donors specifically for this study.

The project was approved and supervised by the Ethics Committee of the Faculty of Medicine at the Universidad Nacional de Colombia, as documented in Act No. 012, dated July 28, 2022.

The procedures for sample collection, storage, transport, and processing were carried out in accordance with the protocols of the Immunology and Translational Medicine Laboratory at the Faculty of Medicine. Data handling remained strictly confidential from the moment of sample collection through to the publication of the results.

### Cell lines and healthy donors

For the transfection of the minigene, HEK293 cells were utilized. These are a cell line derived from human embryonic kidney cells, widely known for their propensity to be transfected. Moreover, specific CD8+ T cell clones targeting the immunogenic epitopes of the CMV pp65 protein and MART-1 were utilized. These clones were derived through limiting dilution. They were generously donated to the I&MT laboratory by Dr. Pedro Romero from the University of Lausanne, Switzerland.

The buffy coats were obtained through an agreement with the Instituto Distrital de Ciencia, Biotecnología e Innovación en Salud (IDCBIS) from their blood bank. This institute continuously conducts blood donations at various locations throughout Bogotá. These blood samples are collected in blood bags containing anticoagulants and transported to the district blood center. Once the sample is obtained, a blood fractionation process is performed to store in the blood bank the fraction that will be used for patient transfusion. The fraction that is not used, in this case the buffy coat, is typically discarded. For this project, this waste was used to obtain PBMCs from healthy donors. Considering that the unused blood fraction from the blood bank samples was involved, it was not necessary to request additional consent from the healthy donors, as they had already signed the institute’s informed consent form, which authorizes the use of this fraction for other types of research. The samples were collected starting on October 1, 2022, and ending on April 30, 2023.

### Cell cultures

Cell cultures of HEK293 cell line was conducted using Roswell Park Memorial Institute 1640 (RPMI 1640) and Dulbecco’s Modified Eagle Medium (DMEM), supplemented with 10% fetal bovine serum (FBS). Monocyte, Dendritic cells (DCs), Peripheral blood mononuclear cells (PBMCs) and T CD8+ clone cultures were performed in serum-free AIM-V medium. Monocytes, PBMCs and clones were centrifuged at 700g for 10 minutes, while HEK293 cells were centrifuged at 200g for 5 minutes. All culture incubations were conducted at 37°C with 5% CO2, unless otherwise indicated in the respective section.

### Isolation of PBMCs from buffy coats of healthy donors

The isolation of PBMCs from healthy donors (between 18 and 30 years old without oncologic, autoimmune, or recent infectious diseases) was carried out using buffy coats from the blood bank, obtained under the agreement with the Instituto Distrital de Ciencia, Biotecnología e Innovación en Salud (IDCBIS).

The buffy coat was distributed into 50 mL polypropylene tubes containing 25 mL of sterile 0.9% saline solution and centrifuged at 700 g for 10 minutes, without brake. This process was repeated 3 or 4 times to remove the anticoagulant. PBMCs were isolated using Lymphoprep™ Density Gradient Medium (STEMCELL™ Technologies), which implements the density gradient separation method. Once separated, cell counting, and viability assessment were performed using a Neubauer chamber with trypan blue staining. The cells were cryopreserved in freezing medium (RPMI-1640 50%, FBS 40%, and dimethyl sulfoxide (DMSO) 10%) and stored at -70°C for 48 hours. After this period, they were transferred to a liquid nitrogen tank for later use in immunological assays. Remaining cellular fractions were discarded following the technical regulations for the disposal of biological waste.

### Minigene design and vector selection

Six viral or tumor antigens were selected to be distributed in the construct: FLU (58-66), CMV (495-503), Mart-1 (26-35), NY-ESO (157-165), Her2-neu (369-377/435-443). In addition to the antigens, a spacer sequence recognized by proteins such as furin protease was added to the construct to separate one antigen from another, allowing them to be hydrolyzed correctly [8]. Codon optimization was performed using the EMBOSS Backtranseq software. The construct was then analyzed with the ProP 1.0 program to determine the optimal order of the antigens that maximizes the probability of furin protease recognizing the spacer and hydrolyzing it specifically. The construct was analyzed using Addgene’s Sequence Analyzer program to determine which restriction sites were present in the minigene sequence. Finally, BamHI and XbaI restriction enzyme sequences were positioned at the ends of the minigene to facilitate cloning of the construct (S1 Table). The minigene was synthesized by Integrated DNA Technologies (IDT). The selected vector was pcDNA 3.1-N-eGFP.

### Plasmid amplification

A bacterial transformation was performed using the E. coli JM109 strain. The bacteria were subjected to heat shock to increase membrane permeability for plasmid entry. Subsequently, they were plated on LB agar containing ampicillin and incubated for 24 hours to select colonies that grew on this selective medium, indicating successful plasmid uptake by the bacteria. The obtained colonies were then transferred to LB agar with ampicillin once again for isolation and further incubation for another 24 hours. After this period, they were inoculated into LB ON (Over Night) medium for storage in 30% glycerol at -80°C to establish a reserve of plasmid-producing bacteria.

### Minigene cloning into the pcDNA 3.1-N-eGFP vector

To clone the minigene into the pcDNA 3.1-N-eGFP plasmid, restriction enzymes BamHI and XbaI (New England Biolabs) were used, which have recognition sites at the ends of the minigene and the multiple cloning sites of the plasmid to create cohesive ends. Subsequently, both the minigene and the plasmid were digested separately for 4 hours with the two enzymes at 37°C, and the enzymes were then inactivated for 20 minutes at 80°C. Following this, ligation was carried out using T4 DNA ligase (Invitrogen) for 1 hour at room temperature. For the extraction of genetic material, the QIAprep Miniprep kit from Qiagen was utilized. The minigene cloning was confirmed by PCR using a forward primer annealing to a section of the plasmid (5’-TAATACGACTCACTATAGGG-3’) and a reverse primer annealing to the end of the minigene (5’-GAGAATATGCGCCATTGTG-3’). Another PCR was performed with a forward primer annealing to the start of the minigene (5’- AACTTGCAGGTATAGGAATC-3’) and a reverse primer annealing to a section of the plasmid located downstream of the minigene (5’-CAGGAAACAGCTATGACCATG-3’). This process confirmed that the minigene was inserted into the plasmid in the correct orientation.

### Production of mature dendritic cells derived from monocytes

Monocyte isolation from buffy coat was performed using a negative selection method employing the RosetteSep™ kit (STEMCELL™ Technologies). To achieve this, the buffy coat was incubated with 50 µ L/mL of the reagent and underwent density gradient separation using Lymphoprep™ reagent. The purity percentage of isolated monocytes was estimated via flow cytometry using the CD14-FITC marker (BioLegend).

For DCs derivation, monocytes were cultured at a concentration of 200,000 cells/well in a 96-well U-bottom plate in AIM-V medium supplemented with 800 U/mL of rhIL-4 (1000 U/mL) and GM-CSF (1000 U/mL) (CellGenix®) and incubated at 37°C for 48 hours. Subsequently, fresh AIM-V medium supplemented with 800 U/mL of GM-CSF and 40 U/mL of IL-4 was added for another 48 hours. Finally, fresh AIM-V medium supplemented with IFN-γ and LPS was added to induce maturation, and incubated for 24-48 hours at 37°C.

### Stimulation of monocyte-derived dendritic cells with different antigen formulations

To stimulate mDCs with different antigen formulations, they were cultured in a 96-well plate with AIM-V medium as mentioned before. Antigens were incorporated into the culture medium as follows: the short peptide (10 μg/mL) was added two hours after initiating the maturation stimulus, whereas both the long peptide (20 μg/mL) and the recombinant protein (30 μg/mL) were incorporated 24 hours before applying the maturation stimulus. As a negative control, a culture of mDCs was not exposed to any antigen format. Specific assays employed short peptides, long peptides, and recombinant proteins corresponding to the pp65 CMV and MART1 antigens, as detailed in each experiment. Table 1 presents the sequences of the short and long peptides used.

**Table 1:** Sequences of long and short peptides utilized in immunological assays.

Antigen	Short peptide sequence^1^	Long peptide sequence^2^
CMV pp65	NLVPMVATV _495-503_	LAR**NLVPMVATV**QGQ _492-506_ [16]
MART-1	ELAGIGILTV _26-35_	GHGHSYTTAE**ELAGIGILTV**ILGVL _16-40_ [17]

^1^Manufactured by 21st Century Biochemicals (USA).

^2^Manufactured by Fundación Salud de los Andes.

The recombinant proteins used to stimulate the DCs were:

- Recombinant Cytomegalovirus pp65 recombinant protein (Abcam Cat # ab43041) – 63kDa
- Recombinant Human MelanA protein (Abcam Cat # ab114312) – 39kDa

### Minigene transfection into HEK293 cells

For transfection, Lipofectamine 3000 from Invitrogen was used. HEK293 cells were cultured to 70-90% confluence in 24 or 48-well plates. In a 1.5 ml polypropylene tube (Eppendorf), 3 µ L of lipofectamine was diluted in 50 µ L of OptiMEM medium. In another tube, 2 µg of DNA and 3 µ L of the p3000 reagent contained in the kit were diluted in 50 µ L of OptiMEM medium, and this was mixed with the former dilution in a 1:1 ratio. The resulting solution was incubated for 30 minutes, and the lipid-DNA complex was subsequently added to the wells.

### Construction of artificial antigen-presenting cells

Artificial antigen-presenting cells (aAPCs) were constructed by transfecting HEK293 cells with cDNAs encoding for HLA-A * 02:01, co-stimulatory molecules CD137-L, CD80, and CD83 along with the minigene, using Lipofectamine 3000. Transfection was performed following the parameters described above. Upon transfection, GFP protein expression in cells was confirmed via fluorescence microscopy. Additionally, the expression of co-stimulatory molecules and GFP was monitored using flow cytometry with anti-HLA-A2-PE, CD80-PE, CD83-APC-Cy7, and CD137L-APC antibodies (BioLegend).

### Donor selection for immunological assays

Processed Buffy coats underwent screening for the HLA-A2 haplotype using flow cytometry. For this, 100 µ L of blood samples were taken, and anti-HLA-A2-PE antibody (BioLegend) was added, followed by incubation at 4ºC in the dark. Subsequently, 1 ml of diluted cell lysis reagent (BD FACS Lysing solution) was added, incubated for 5 minutes at room temperature, and washed with PBS 5% FBS at 700g for 7 minutes. From each positive sample, 125μL of buffy coat were deposited onto Protein Saver 903 Whatman® cards. The cards were sent for high-resolution HLA-A typing (2x Exons 2 and 3) to Histogenetics LLC. Donors carrying the HLA-A * 02:01:01 haplotype, whether homozygous or heterozygous, were selected for the immunological assays.

### 9-Day culture of total PBMCs for expansion of antigen-specific T cells from healthy donors

To obtain antigen-specific T cells against the antigens encoded in the minigene, PBMCs obtained from HLA-A * 02:01 healthy donors were used in a 9-day culture system employing with IL-21 (30 ng/mL), IL-7 (5 ng/mL), and IL-15 (5 ng/mL) (CellGenix®) to stimulate the expansion of antigen-specific CD8+ T cells. This methodology was standardized at the I&MT [4,18].

For this purpose, 1x106 cells/well of total PBMCs were cultured in U-bottom 96-well plates in AIM-V medium supplemented with IL-21 on day 0 and pulsed (stimulated) or not (unstimulated) with 10 μg/mL of CMV pp65495-503 (NLVPMVATV) peptide (21st Century Bio-chemicals, Inc, Marlborough, Massachusetts, USA). The plate was incubated for 72 hours under normal culture conditions, and the culture medium was changed every 2 or 3 days with fresh AIM-V supplemented with IL-7 and IL-15. On day 9, the cells were evaluated by flow cytometry to detect antigen specific CD8+ T cells or were used for the evaluation of intracellular cytokine production or expression of activation and exhaustion markers.

### Identification of antigen specific T cells by flow cytometry using tetramer staining

The identification of antigen-specific CD8+ T cells was conducted by labeling with HLA-A * 02:01 tetramers (Tetramershop) loaded with the target antigens (short peptides from pp65 CMV, MART-1). Empty tetramers were individually loaded with the corresponding peptides at a concentration of 200 µ M for 30 minutes at 4°C.

After 9 days of culture, PBMCs were harvested from the culture plate and washed at 700 g for 7 minutes. Subsequently, cells were labeled for 15 minutes at 37°C with 5ul of the assembled tetramer with the antigens of interest, then they were stained with Zombie Aqua (BioLegend) (1:1000 in PBS) for 30 minutes at room temperature and washed at 700 g for 7 minutes in PBS with 2% FBS. PBMCs were stained with CD3-PB and CD8-PE-Texas Red, resuspended in 100 µ L of PBS, and analyzed by flow cytometry. Each sample reading comprised a minimum of 30,000 events per experiment, using the FACS Aria IIIu System at the Universidad Nacional de Colombia – Facultad de Medicina, and the results were analyzed with FlowJo v10.0.7 software (Treestar Inc). The graphics were generated using Prism v8 software (Graph Pad).

### Measurement of intracellular cytokines by flow cytometry

After 9 days of culture, PBMCs were harvested from the culture plate and washed at 700 g for 7 minutes. They were then co-cultured in a 1:1 ratio with: i) thawed autologous PBMCs stimulated with CMV short peptide or unstimulated PBMCs; ii) HEK293 cells transfected with Co-stimulatory Molecules and pcDNA3.1-N-EGFP-Minigene encoding a fusion protein GFP-Minigene, HEK293 cells transfected with GFP-Minigene, HEK293 cells transfected with GFP and untransfected HEK293 cells; iii) autologous mDCs stimulated with short CMV peptide, long CMV peptide or recombinant pp65 CMV protein, and autologous mDCs unstimulated. Antigen specific CD8+ T cell clones (CMV + , Mart-1+) were thawed one day prior to use to allow for overnight recovery in AIM-V medium. The next day, they were co-cultured at a 6:1 ratio (antigen-presenting cells:clone) with the same cells described before.

The presenting PBMCs were thawed and pulsed for 16 hours with the peptides of interest at a concentration of 10 µg/mL and stained with CFSE (1:5000 dilution) or CellTrace™ Far Red (1:1000 dilution) to differentiate them from the PBMC population derived from the 9-day lineage or from the antigen specific CD8+ T cell clones during sample reading by flow cytometry.

At 1.5 hours post-co-culture initiation, Brefeldin A 1X (BioLegend) was introduced to each well, followed by a 4.5-hour incubation period. Subsequently, cells were harvested, and their viability was assessed using Zombie Aqua. Surface markers CD3-PB and CD8-PE-Texas Red (BioLegend) were then stained. Cells were further fixed and permeabilized for intracellular staining using the IntraStain kit from Dako, followed by staining of intracellular cytokines with anti-TNF-α-FITC and IFN-γ-PE antibodies (BioLegend). Flow cytometry analysis was conducted with a minimum of 30,000 events per experiment.

### Evaluation of activation and exhaustion phenotypes in CD8+ T cells

PBMCs from the cell line were collected on day 9 or the antigen specific CD8+ T cell clones were thawed one day prior to use and subsequently, they were co-cultured at a 1:1 or 6:1 ratio with the following: i) thawed autologous PBMCs stimulated with CMV short peptide or unstimulated PBMCs; ii) HEK293 cells transfected with Co-stimulatory Molecules and pcDNA3.1-N-EGFP-Minigene encoding a fusion protein GFP-Minigene, HEK293 cells transfected with GFP-Minigene, HEK293 cells transfected with GFP and untransfected HEK293 cells; iii) autologous mDCs stimulated with short CMV peptide, long CMV peptide or recombinant pp65 CMV protein, and autologous mDCs unstimulated.

After 24 hours of culture, PBMCs or antigen specific CD8+ T clones were harvested, and their viability was assessed using Zombie Aqua staining. Surface markers CD3-PB, CD8-PE/Texas-Red, CD137-PE-Cy5, CD25-APC-Cy7, CD69-BV-650, OX40-BV-711, CTLA-4-PE-Cy7, PD-1-PerCP-Cy5.5, LAG-3-BV786, CD62L-AF700, and CD45RO-FITC were also labeled. Additionally, the CD154-PE marker was added to the culture medium in the corresponding wells at the time of co-culture establishment. Each sample reading consisted of a minimum of 30,000 events per experiment.

### Statistical analysis

Statistical analysis was conducted using GraphPad Prism v8 software. Between-group comparisons were assessed using the Mann-Whitney and Kruskal-Wallis tests, as appropriate. Significant differences are depicted in the graphs, and the corresponding p-values are provided in the figure legends.

## Results

### Construction of artificial antigen presenting cells (aAPCs) using HEK293 cells

HEK293 cells (Human Epithelial Kidney) are epithelial-origin cells derived from human embryonic kidney cells. They are widely recognized for their utility in viral vector replication and production; these cells exhibit notable traits such as high transfection efficiency and accurate translation of proteins encoded by transfected DNA.

Flow cytometry analysis of the immunophenotype of HEK293 cells revealed the expression of several markers, including the co-stimulatory molecule CD80, as well as HLA-DR and CD14, commonly found on the membrane of myeloid-derived antigen-presenting cells (APCs) like monocytes and macrophages. Notably, the expressions of CD83 and CD40, which are characteristic of professional APCs such as dendritic cells (DCs), were absent (S1 Fig). Additionally, staining with the BB7 antibody, which recognizes a significant subset of HLA A * 02:01 alleles (referred to as HLA-A2), demonstrated notable surface staining of HEK293 cells (S2 Fig).

This surface molecule expression pattern led to the use of HEK293 cells as a platform for investigating their usefulness in engineering “artificial” APCs (aAPCs). HEK293 cells were transfected using Lipofectamine 3000 with the pcDNA3.1-N-EGFP-Minigene plasmid, which encodes a fusion protein comprising GFP and a minigene containing well-known viral and tumor-derived A2-restricted epitopes (referred from now on as GFP-Minigene). Additionally, cells were transfected with the pcDNA3.1-N-EGFP plasmid, which encodes GFP alone (referred to as GFP) and the GFP expression levels were measured via flow cytometry.

S3 Fig shows the transfection results, where an expression of GFP was observed when cells were transfected with GFP alone and with GFP-Minigene. However, cells transfected with GFP-Minigene exhibited lower GFP expression compared to those transfected with GFP alone, suggesting that the presence of the minigene may exert a toxic effect on GFP expression.

**Fig 1:**
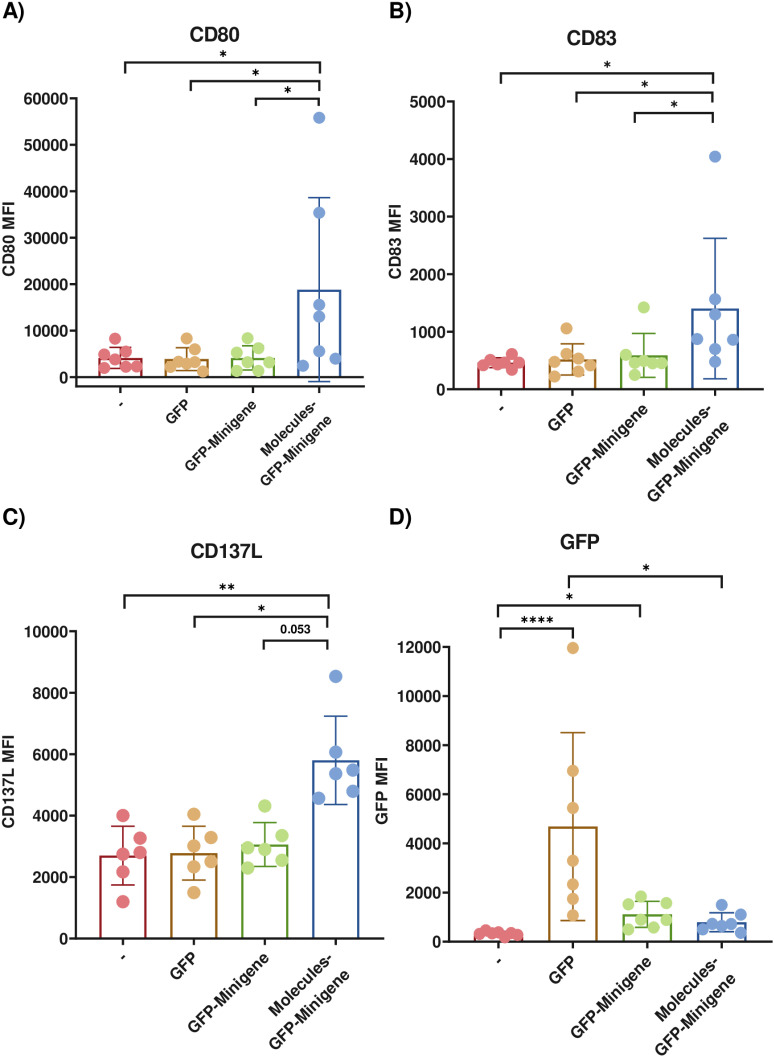
Expression of co-stimulatory molecules in HEK293 cells co-transfected with different plasmids. HEK293 cells were simultaneously co-transfected with plasmids encoding CD80, CD83, CD137L with the pcDNA 3.1 - N-eGFP-Minigene plasmid (Molecules-GFP-Minigene). The expression of each marker was measured in cells co-transfected with all markers and the minigene (Molecules-GFP-Minigene), cells co-transfected solely with the minigene (GFP-Minigene), cells transfected only with the GFP plasmid (GFP), and non-transfected cells. The bars indicate the mean +  SEM for 6 replicates. Statistical analysis was performed using Kruskal-Wallis tests with a p-Value <  0.05. Data are representative of 6 replicates.

To optimize antigen presentation to CD8 T-cells, we co-transfected HEK293 cells with the minigene and plasmid encoding the co-stimulatory molecules CD80, CD83, and CD37L, which are typically expressed by professional antigen-presenting cells. To evaluate the impact of transfection on the expression of these molecules, the plasmids were co-transfected with the GFP. We found a statistically significant increase in CD80, CD83, and CD137L expression in cells co-transfected with plasmids encoding these molecules along with GFP-Minigene (Fig 1 A - C). Regarding GFP expression, as observed before, there was a statistically significant difference in cells transfected solely with GFP (Fig 1D). However, GFP expression was also significantly higher in cells transfected with the GFP-Minigene construct compared to non-transfected cells (Fig 1D). These results support the use HEK293 cells transfected as antigen-presenting cells in subsequent functional assays with CD8+ T cells.

### Screening of PBMCs from HLA-A * 0201 healthy donors having CD8 T cell precursors for CMV

To identify CD8+ T cell precursors to the short epitope of pp65 CMV, we screened PBMCs from HLA-A02:01 healthy donors. PBMCs were cultured in a round-bottom 96-well plate for nine days with IL-21, IL-7, and IL-15. On day nine, the non-adherent fraction of PBMCs was collected and stained with a tetramer loaded with the corresponding peptide. To standardize this methodology, a CMV peptide (a 9-amino acid epitope of the pp65 protein, recognized by HLA-A * 0201-restricted CD8+ LT cells) was used.

**Fig 2:**
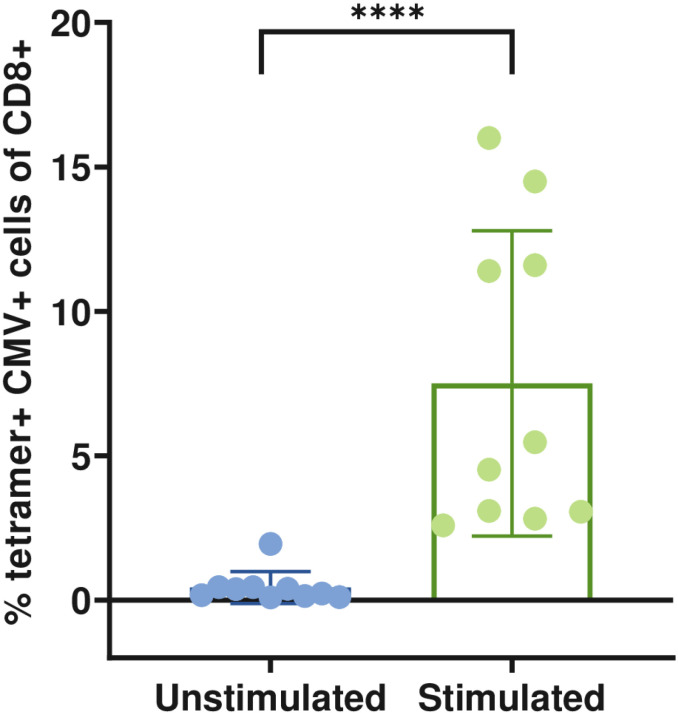
Identification of CMV-specific CD8+ LT cells for the HLA-A * 0201 epitope. Representative bar graph showing the expansion of CMV-specific CD8 + LT cell populations in 10 evaluated donors after 9 days of stimulation with CMV peptide and IL-21, IL-7, and IL-15 (Stimulated), compared to unstimulated cells (Unstimulated). The analysis was performed using the Mann-Whitney test (n =  10); p < 0.05. Error bars represent mean +  SEM.

Out of the 17 screened healthy HLA-A * 0201 donors, 10 exhibited a population of CMV pp65-specific CD8+ T tetramer-positive cells (S2 Table). To confirm the specificity of these cells, we used two tetramers loaded with the same antigen but with two different fluorochromes. Fig 2 shows the mean percentage of significant expansion of a CMV-specific CD8+ T cell population from 10 screened donors in response to peptide and cytokine stimulation for nine days *in vitro*, as described in the Materials and Methods section.

### HEK293 cells transfected with the minigene efficiently stimulate polyfunctional CD8+ T cell populations in PBMCs from healthy donors

To evaluate the ability of minigene-transfected HEK293 cells to stimulate antigen-specific CD8 + T cells, we assessed the intracellular production of IFN-γ and TNF-α by CD8 + T cells in PBMCs from healthy donors with detectable CMV-specific CD8 + T cells precursors. PBMCs underwent a 9-day stimulation protocol, initially by pulsing them with the short CMV peptide in AIM-V medium supplemented with IL-21, IL-7, and IL-15. Subsequently, on day 9, cultures were re-stimulated for 6 hours with HEK293 transfected with the minigene as aAPCs in the presence of brefeldin A. Following this incubation period, cells were harvested, and CD8 + T cells expressing these two intracellular cytokines were quantified using flow cytometry.

**Fig 3:**
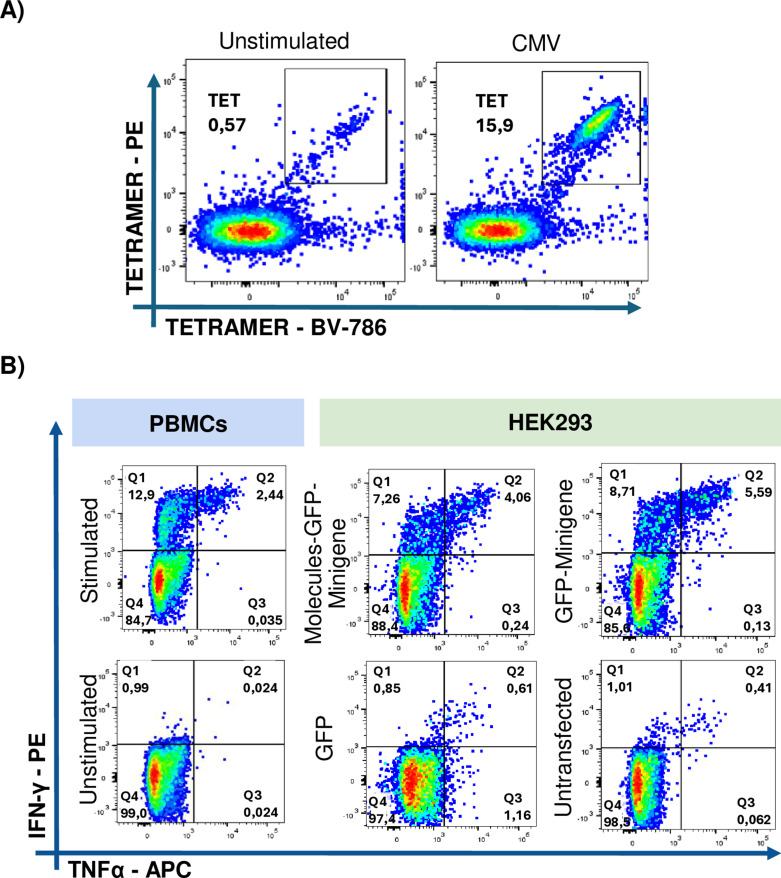
Intracellular cytokine expression by CD8+ T cells from 042 donor upon restimulation with aAPCs. A) The expansion of the tetramer-positive CD8+ T cell population achieved after the stimulation for nine days of PBMCs with the short pp65 peptide from CMV was compared to the expansion achieved in cultures of non-stimulated cells. B) Intracellular cytokine expression by CD8+ T cells cocultured with HEK293 cells transfected with Molecules-GFP-Minigene; the GFP-Minigene and with GFP alone or with non-transfected HEK293 cells. Autologous PBMCs pulsed with the short pp65 peptide from CMV were used as a positive control, while unstimulated PBMCs and non-transfected HEK293 cells served as negative controls.

The first donor analyzed was 042, who is homozygous for HLA-A * 02:01. After nine days, the expansion of the CMV-positive CD8 + T cells tetramer population was assessed using HEK293 cells as aAPCs, revealing a proportion of 15.9% CMV + CD8 + T cells (Fig 3A). Notably, cotransfection with plasmids encoding the co-stimulatory molecules resulted in 1.4-fold lower population of IFN-γ+ TNFα+ CD8+ T cells compared to HEK293 cells transfected with the GFP-Minigene construct alone. Similarly, the IFN-γ+ CD8+ T cell population stimulated by GFP-Minigene-transfected cells was 1.2 times greater than that observed with Molecules-GFP-Minigene-transfected cells. No IFN-γ- TNF-α+ CD8+ T cell population was detected (Fig 3B). Cytokine production was dependent on antigen presentation, as co-cultures with HEK293 cells lacking minigene transfection failed to elicit expansion of CD8+ T cells specific for the encoded epitopes (Fig 3B).

**Fig 4:**
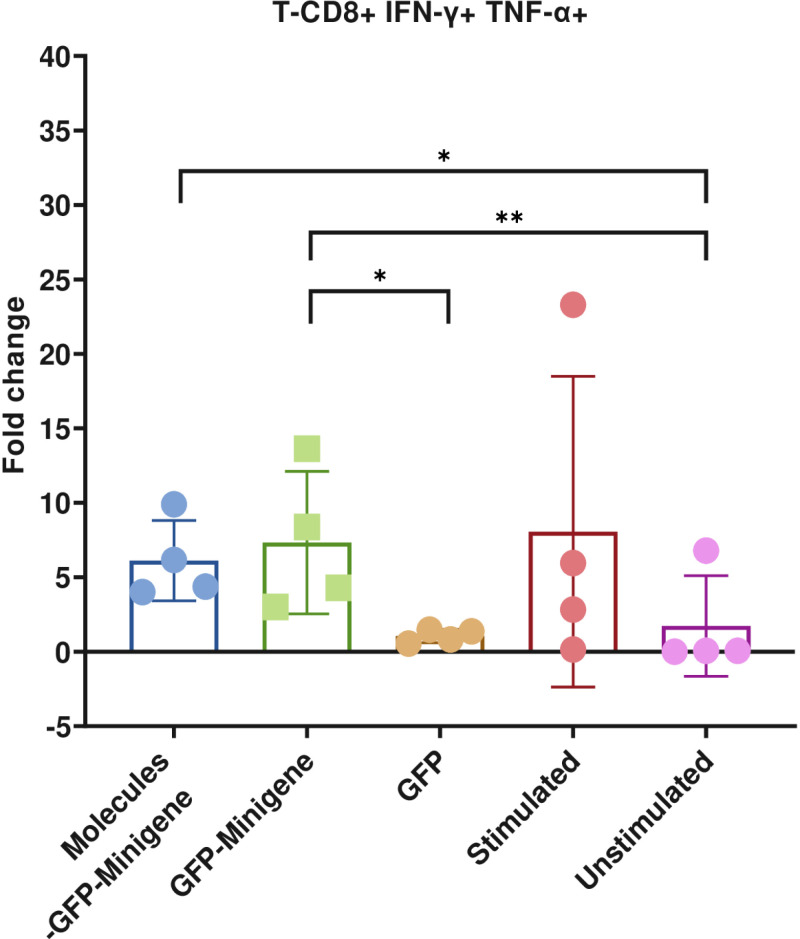
Intracellular cytokine expression in CD8+ T cells from healthy donors cocultured with HEK293 cells as aAPCs. Bars representing the fold change of populations of IFN-γ+ TNFα+ CD8+ T cells from 4 healthy donors cocultured with HEK293 cells transfected with Molecules-GFP-Minigene, GFP-Minigene, GFP alone, untransfected cells, compared to the positive control (PBMCs stimulated with the CD8 epitope from CMV) and the negative control (unstimulated PBMCs). Statistical analysis of the groups was conducted using the non-parametric Kruskal-Wallis test, n = 4, (p < 0.05). The bars represent independent experimental replicates +  SEM.

As sum up for these experiments, Fig 4 illustrates the fold change in IFN-γ+ TNFα+ CD8 + T cells derived from the PBMCs of four healthy donors following stimulation with transfected HEK293 cells (S3 Table). The analysis revealed no significant differences between cells cocultured with Molecules-GFP-Minigene-transfected cells and those with GFP-Minigene-transfected cells. Additionally, S4 Fig presents data for the IFN-γ+ TNFα- CD8+ T cell population, confirming that IFN-γ expression in T cells is antigen-specific. Significant differences were observed when comparing cells transfected with Molecules-GFP-Minigene and GFP-Minigene to those transfected only with GFP. However, no significant differences were detected between cells transfected with Molecules-GFP-Minigene and those with GFP-Minigene, indicating that co-stimulatory molecule transfection does not enhance intracellular cytokine production in healthy donors. Furthermore, no significant differences were noted between the CD8 + T cells specific to CMV cocultured with minigene-transfected HEK293 (acting as aAPCs) and those stimulated with peptide-pulsed PBMCs (used as positive controls). S3 Table provides a summary of the fold change in IFN-γ +  TNF-α +  T cells and IFN-γ +  TNF-α – T cells cultured with HEK293 transfected cells, observed in the different intracellular cytokine assays.

### Antigen-specific CD8+ T cell clones exhibit a cytotoxic profile when co-cultured with HEK293 cells transfected with the minigene

After using transfected HEK293 cells as aAPCs to stimulate T cells precursors present in PBMCs from healthy donors, we extended our approach to antigen-specific CD8 + T cell clones for CMV and MART-1. This allowed us to evaluate the potential impact of co-stimulatory molecules on a larger population of antigen specific CD8+ T cells.

**Fig 5:**
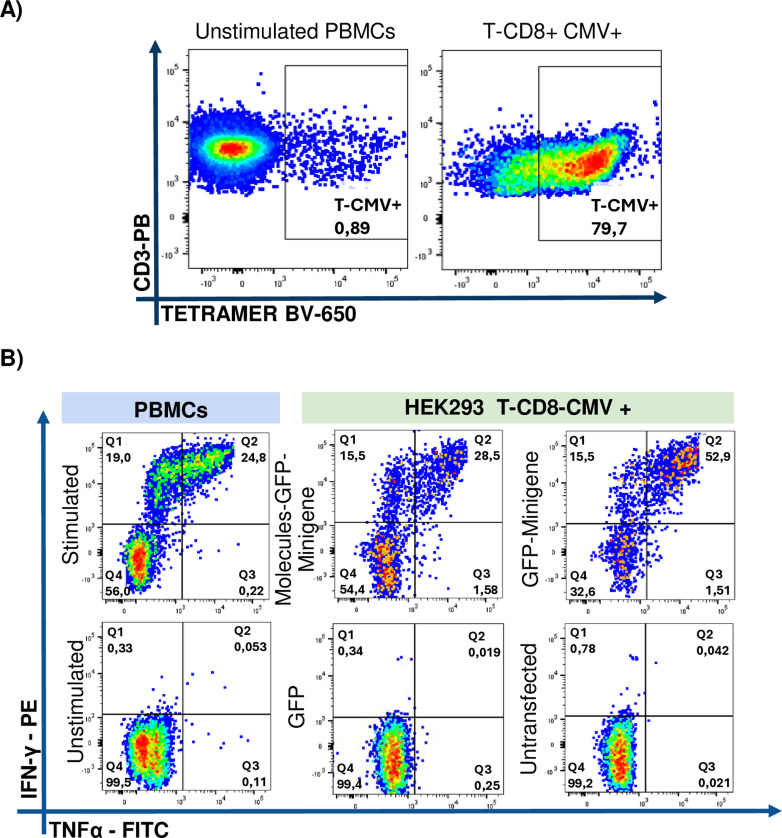
Expression of IFN-γ +  TNF-α +  by CD8-CMV + in co-culture with APCs transfected with Minigene and co-stimulatory molecules. A) Tetramer-positive population of CMV-specific CD8 + T clones compared to unstimulated PBMCs. B) Intracellular cytokine expression of CMV-specific CD8 + T cells co-cultured with HEK293 cells transfected with Molecules-GFP-Minigene, GFP-Minigene, GFP alone and untransfected HEK293 cells. Autologous PBMCs pulsed with the short peptide pp65 of CMV were used as positive controls, while unstimulated PBMCs served as negative controls.

For these experiments, antigen-specific T cell clones were thawed one day before use and were left in AIM-V culture medium overnight. The next day, intracellular cytokine evaluation was carried out as mentioned earlier. Following staining with specific CD8+ T tetramers against the CMV epitope, a population close to 80% of CMV-specific CD8+ T cells was found (Fig 5A).

When these cells were co-cultured with aAPCs co-transfected with plasmids encoding the co-stimulatory molecules CD80, CD83, CD137L, along with the GFP-Minigene fusion (Molecules-GFP-Minigene), a 1.19-fold increase in IFN-γ+ cells (15.5%) was observed compared to co-cultures with HEK293 cells transfected with GFP-Minigene (13%). In contrast, the population of IFN-γ+ TNF-α+ cells was higher in co-cultures with GFP-Minigene-transfected APCs (52.9%) than in those with Molecules-GFP-Minigene-transfected aAPCs (28.5%).

While the TNF-α+ CD8+ T cell population was smaller compared to the two populations previously described, an increase in TNF-α expression is observed in cocultures of T cell clones with HEK293 cells transfected with either Molecules-GFP-Minigene or GFP-Minigene, compared to peptide-pulsed PBMCs used as positive control APCs. An important aspect to note is that when coculturing CD8+ T cells with non-transfected HEK293 cells, no expression of any of these cytokines was observed. (Fig 5B).

**Fig 6:**
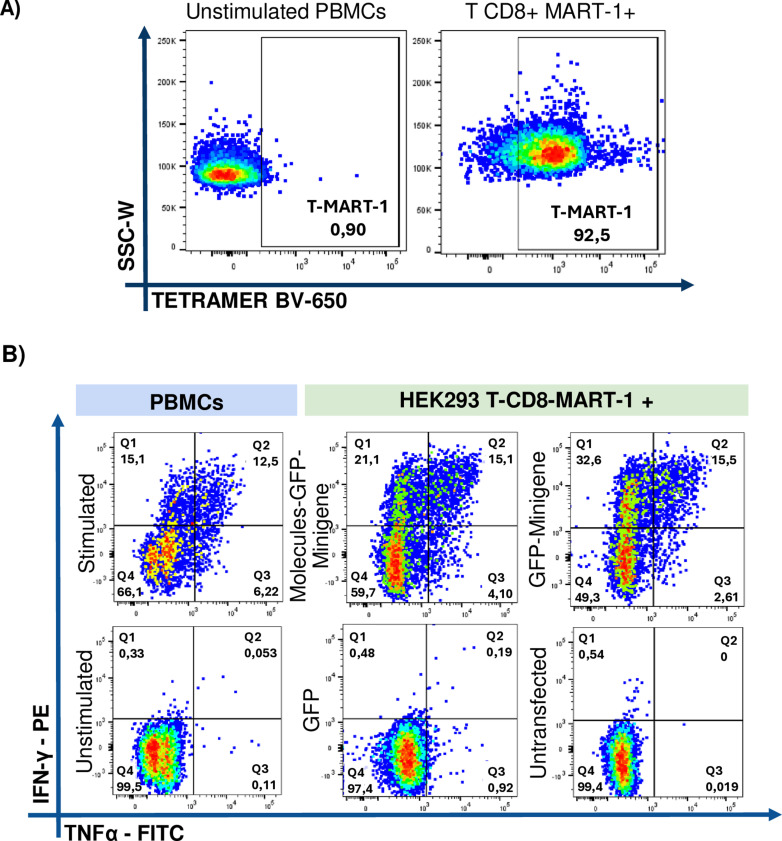
Expression of IFN-γ +  TNF-α +  by CD8-Mart-1 + in co-culture with APCs transfected with Minigene and co-stimulatory molecules. A) Tetramer-positive population of T CD8 + Mart-1 + clones compared to unstimulated PBMCs. B) Intracellular cytokine expression of T CD8 +  Mart-1 +  cells co-cultured with HEK293 transfected with Molecules-GFP-Minigene, with GFP-Minigene, GFP only and untransfected HEK293 cells. Autologous PBMCs pulsed with the short peptide of Melan-A were used as positive control, and unstimulated PBMCs as negative control.

To further investigate antigen presentation by transfected HEK293 cells, CD8 + T cell clones specific for MART-1 were analyzed (Fig 6A). Following the protocol mentioned for CMV clones, we observed that APCs transfected with Molecules-GFP-Minigene stimulate a smaller population of IFN-γ+ (21.1%) than those transfected with GFP-Minigene alone (32.6%). The TNFα+ population followed a similar trend (4.10% vs 2.61%), unlike the observations made with T CD8+ CMV + cells. Furthermore, the IFN-γ+ population in both cases were greater than that observed when using peptide-pulsed PBMCs (positive control). The double-positive population remains similar in both conditions (approximately 15%) and was higher than that observed in the positive control (12.5%) (Fig 6B, upper panel). This suggests that HEK293 cells exhibit a higher efficiency of presentation than APCs present in PBMCs. However, as observed in previous experimental results measuring the expansion of antigen-specific T CD8+ , no clear benefit could be attributed to the co-transfection of HEK293 cells with a plasmid encoding co-stimulatory molecules when used as aAPCs.

Furthermore, no nonspecific response was found with the use of transfected HEK293 cells since no reactive population of CD8+ T cells was evident in the co-culture with GFP-transfected APCs or non-transfected APCs (Fig 6B, lower panel).

**Fig 7:**
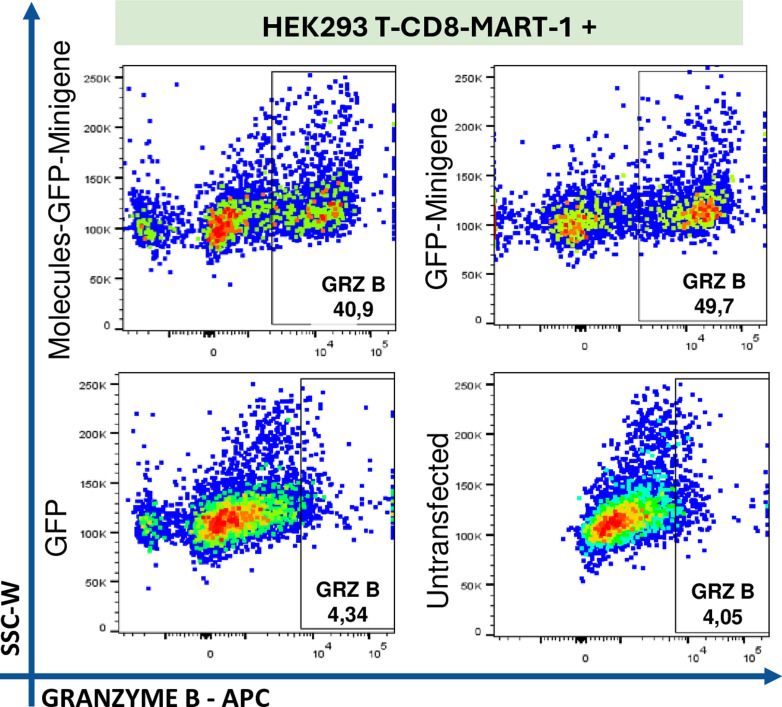
Evaluation of Granzyme B expression in CD8+ Mart-1 + co-cultured with transfected HEK293 cells. The population of T CD8+ Mart-1 + cells co-cultured with HEK293 cells transfected with Molecules-GFP-Minigene, GFP-Minigene and GFP only expressing Granzyme B intracellularly assessed by flow cytometry. This was compared with CD8+ Mart-1 + co-cultured with untransfected HEK293 cells.

Finally, the cytotoxic capacity of Mart-1-specific CD8+ T cells was evaluated by assessing Granzyme B production. A Granzyme B + CD8+ T cell population was identified, comprising 40% of those co-cultured with HEK293 cells transfected with the Molecules-GFP-Minigene and 49% of CD8+ T cells co-cultured with cells transfected with GFP-Minigene. These results suggest that the cytotoxic phenotype of this clone, as evidenced in these experiments, was antigen-dependent, as Granzyme B production was 6.9-fold and 9.3-fold higher in co-cultures with the minigene-transfected HEK293 cells compared to GFP-transfected or non-transfected cells, respectively (Fig 7).

### Stimulated PBMC-derived CD8+ T cells with artificial antigen-presenting cells express markers of activation and exhaustion

The donor 042, who was used to assess intracellular cytokine production, was employed to examine the immunophenotype of T CD8+ in co-culture for 24 hours with aAPCs. Activation immunophenotype was evaluated by the expression of activation markers CD25, CD137, CD154, and CD69, while exhaustion immunophenotype was assessed through the expression of CTLA-4, PD-1, and LAG-3 using flow cytometry. Three replicates were performed, followed by a Cluster Identification, Characterization, and Regression (CITRUS) analysis. CITRUS analysis is a multidimensional analysis system for flow cytometry data designed to discover populations with statistically significant biological signatures. This analysis compared co-cultures with HEK293 cells transfected with Molecules-GFP-Minigene versus GFP-Minigene, aiming to determine whether co-transfection with co-stimulatory molecules influences CD8+ T cell activation and phenotype.

Three populations of CD3+ CD8+ cells showed statistically significant differences according to the Prediction Analysis Microarray (PAM) model, a predictive tool used to identify associations between the properties (phenotype of the cells) of the calculated clusters and a specific response. The phenotype of all cell groups regarding activation and exhaustion markers are shown in S5A Fig, whereas yellow histograms correspond to high, green correspond to medium, and blue to low or no marker expression. In S5B Fig, the populations highlighted in red (particularly 291107, 291100, and 291110) demonstrated a significant difference.

**Fig 8:**
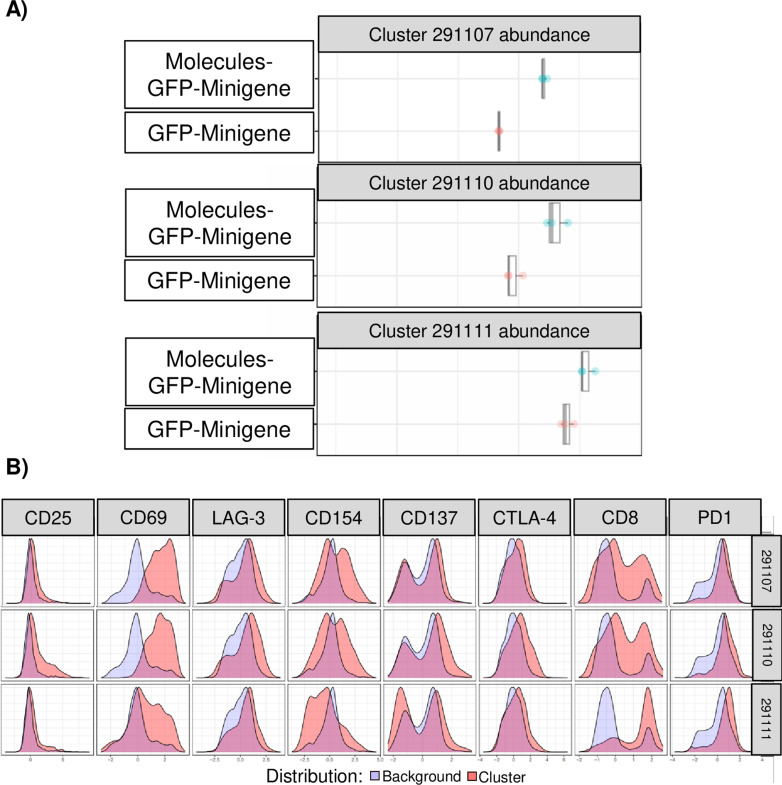
Phenotype of activation and exhaustion of CD8 T lymphocytes present in PBMCs from donor 042 using CITRUS. A) Box and whisker plot of the abundance of each CD3+ CD8+ population present in PBMCs co-cultured with HEK293 cells transfected with Molecules-GFP-Minigene (blue) and CD3+ CD8+ present in PBMCs co-cultured with HEK293 cells transfected with GFP-Minigene (red). B) Expression histograms of markers in populations identified in panel **C** (red), comparing background expression (blue).

Fig 8A shows the abundances of these three populations on a base-10 logarithmic scale. It is observed in box plots that CD8+ T cells co-cultivated with HEK293 cells transfected with Molecules-GFP-Minigene (blue) express more activation and exhaustion markers than CD8+ T co-cultivated with HEK293 cells transfected with GFP-Minigene (red), although it is important to note that in two out of three populations (291107 and 291110), lymphocytes are also activated in the absence of the co-stimulatory molecules.

In Fig 8B, the phenotype of these three CD8 + T populations is shown, the first (291107) being CD3 + CD8 + CD69 + LAG-3 + CD154 + , the second (291100) CD3 + CD8 + CD25 + CD69 + LAG-3 + CD154 + CD137 + CTLA-4 + , and the third (291110) CD3 + CD8 + CD69 + LAG-3 + PD-1 + . These populations exhibited differential expressions of the activation and exhaustion markers mentioned. Interestingly, the LAG-3 marker is present in all three clusters. According to the literature, this is a marker expressed after the expression of PD-1 or CTLA-4. Additionally, all three populations expressed CD69, which is an early activation marker of T lymphocytes. It is interesting that the results of the automated analysis allowed demonstrating that, as expected, the use of co-stimulatory molecules induces greater stimulation of lymphocytes.

The phenotypes of the remaining populations outlined in S5B Fig are shown in S6 Fig These clusters predominantly exhibited a CD3 + CD8-CD69 + CD154 + PD-1 + phenotype, which were primarily stimulated by HEK293 cells transfected with Molecules-GFP-Minigene. The marker expression tree revealed that nearly all CD8– clusters displayed moderate to high expression levels of CD154. The preferential stimulation of these populations by the co-culture of T lymphocytes with HEK293 cells transfected with genes encoding co-stimulatory molecules, alongside minigenes encoding CD8 epitopes, suggests an advantage in using these cells for monitoring the phenotype of CD8 + precursor cells epitope-specific present in PBMC samples of healthy donors.

### Transfected HEK293 cells process and present antigens more efficiently than mDCs stimulated with the full protein antigen

In addition to the results obtained from healthy donors, the ability of autologous DCs pulsed with short and long peptides and the recombinant protein (See specifications in Table 1) containing the HLA-A * 0201 epitope of CMV to stimulate populations of cytokine-producing intracellular CD8 + T cells was evaluated. This was compared with the performance of HEK293 cells transfected with minigene and co-transfected with plasmids encoding co-stimulatory molecules. Monocytes obtained as described in the methodology were cultured in AIM-V medium supplemented with GM-CSF and IL-4 for 5 days, adding fresh medium when necessary to derive DCs. On this day, immature DCs (iDCs) were pulsed with the long peptide and the complete pp65 protein of CMV and incubated for 24 hours, then fresh AIM-V medium supplemented with LPS and IFN-γ was added to achieve DC maturation, and 2 hours later, they were pulsed with the short peptide.

Once maturation of DCs (mDCs) was achieved, they were co-cultured for 6 hours with the non-adherent fraction of PBMCs that had been stimulated with the short peptide of CMV nine days before, in the presence of Brefeldin A. Then cells were collected and evaluated by flow cytometry for the expression of surface markers CD3 and CD8, and intracellular production of IFN-γ and TNF-α.

Autologous PBMCs stimulated with the short peptide of CMV were used as positive controls, while unstimulated autologous PBMCs, unstimulated mDCs, HEK293 cells transfected with GFP, and untransfected HEK293 cells were used as negative controls.

**Fig 9:**
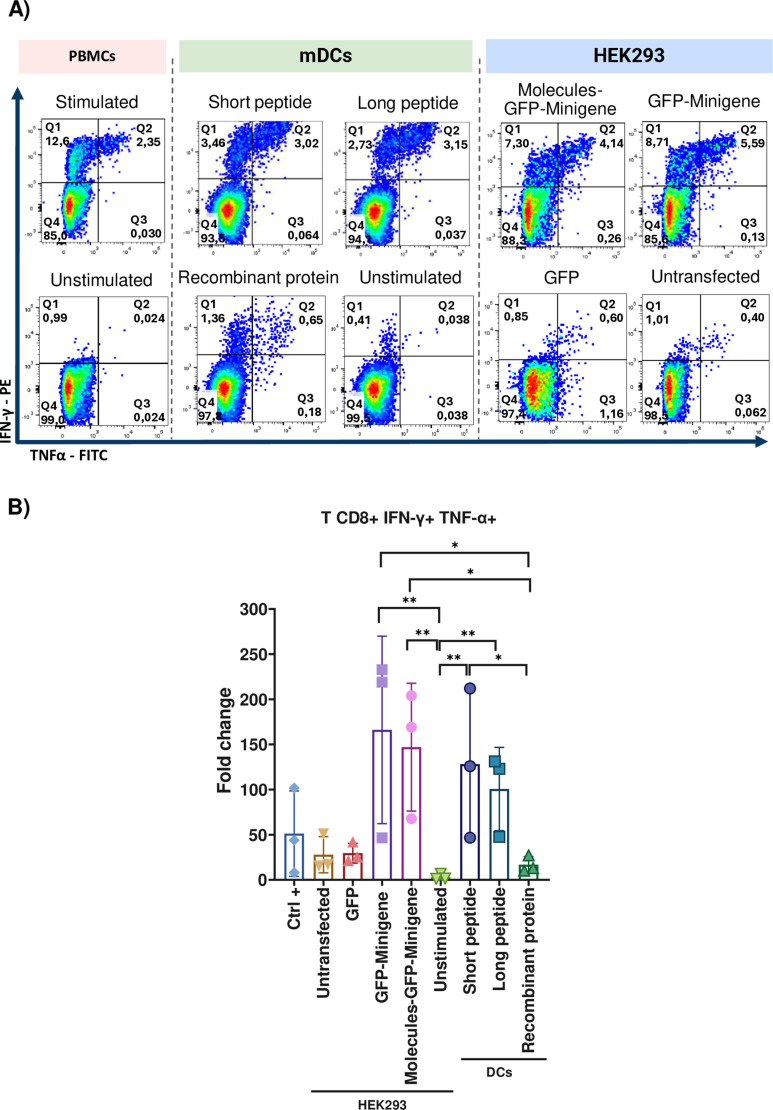
Expression of intracellular cytokines by CD8+ T cells from healthy donors co-cultured with autologous or artificial APCs. A) Expression of intracellular cytokines by CD8+ T cells from donor 042 co-cultured with autologous mDCs (green box) stimulated with the short, long, and pp65 recombinant protein of CMV peptides and with unstimulated mDCs. They were also co-cultured with HEK293 cells (blue box) transfected by lipofectamine with Molecules-GFP-Minigene, with GFP-Minigene, only with GFP, and with untransfected cells. Autologous PBMCs pulsed with the short peptide of CMV were used as positive control (red box), and unstimulated PBMCs were used as negative control. B) Representative bar graphs of fold changes in populations of CD8+ T cells producing IFN-γ+ TNF-α+ from 3 healthy donors co-cultured with the cells mentioned in section A of the figure. Statistical analysis of groups was performed using the non-parametric Kruskal-Wallis test, n = 3, (p < 0.05). Bars represent independent experimental replicates +  SEM.

In donor 042, it was observed that HEK293 cells transfected with the GFP-Minigene fusion protein and HEK293 cells co-transfected with Molecules-GFP-Minigene stimulated a population of polyfunctional CD8+ T cells almost nine times greater than the population of CD8 + T cells stimulated by mDCs pulsed with the complete pp65 protein of CMV (Fig 9A). Furthermore, the populations of single-cytokine-producing CD8+ T cells stimulated by co-culture with transfected HEK293 cells were also larger than those stimulated by mDCs pulsed with short and long peptides of CMV (Fig 9A). It is noteworthy that the presence of untransfected HEK293 cells or cells transfected only with the plasmid encoding GFP did not stimulate populations of non-specific CD8 + T cells.

Fig 9B displays polyfunctional CD8+ T cell populations from three healthy donors, where similar observations were made; it was found that co-culture with HEK293 cells transfected with GFP-Minigene and with Molecules-GFP-Minigene stimulated significantly larger populations of IFN-γ+ TNFα+ CD8+ T cells than those stimulated with mDCs pulsed with the protein. Additionally, no significant differences were found between the populations of double-positive CD8 + T cells stimulated by HEK293 cells as aAPCs and mDCs pulsed with short and long peptides. The population of TNFα+ CD8+ T cells and IFN-γ+ CD8+ T cells are shown in S7 Fig.

Summing up these results, it can be stated that the presentation efficiency of mDCs was not significantly higher than aAPCs, and that HEK293 cells transfected with the minigene favor the natural processing of epitopes, in this case, CMV, over the processing of the complete protein by mDCs.

**Fig 10:**
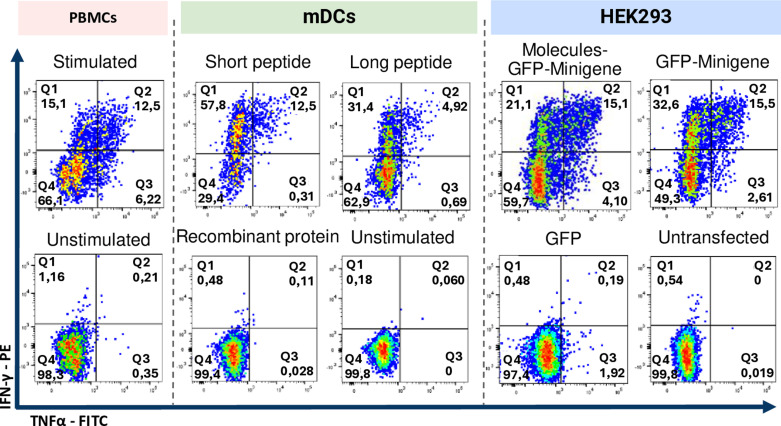
Intracellular cytokine expression of Mart-1+ CD8+ T cells cocultured with autologous or artificial APCs. Intracellular cytokine expression of Mart-1 + CD8+ T cells cocultured with autologous mDCs (green box) co-cultured with short and long peptide, and Mart-1 complete protein, and with unstimulated mDCs. They were also co-cultured with HEK293 cells (blue box) transfected with Molecules-GFP-Minigene, GFP-Minigene, GFP only, and untransfected cells. Autologous PBMCs pulsed with MART-1 short peptide were used as positive control (red box), and unstimulated PBMCs as negative control.

After using PBMCs from healthy donors to compare the epitope processing and presentation capacity of aAPCs with autologous mDCs to stimulate cytokine-producing T precursors, it was decided to use CD8 + antigen-specific clones for MART-1 to evaluate in a more refined model which APC was more efficient. The clones were thawed one day before use, left in AIM-V culture medium overnight, and the next day, intracellular cytokine evaluation was conducted as mentioned earlier. These were co-cultured with HEK293 cells transfected as described earlier and with mDCs pulsed with short, long, and pp65 recombinant protein of Mart-1 (Fig 10). It was evidenced that HEK293 cells transfected with Molecules-GFP-Minigene and GFP-Minigene stimulated a larger, statistically significant population of IFN-γ+ TNF-α+ CD8 + T cells compared to that stimulated by mDCs pulsed with the recombinant protein (Fig 10). Like the case of PBMCs from healthy donors, mDCs were not significantly better antigen-presenting cells than HEK293 cells in regards of short and long peptide stimuli.

## Discussion

Identifying tumor antigens of immunological significance for cancer immunotherapy heavily relies on detecting tumor-reactive T cells. This process is hampered by several factors, including (i) the low number of T cell precursors in the repertoire that recognize them [19], and (ii) tolerance mechanisms in T cells promoted by the tumor microenvironment [20]. Over time, various strategies have been employed to expand antigen-specific T cells to detectable levels using assays such as cell proliferation, measurement of cytolytic activity, and intracellular cytokine production [21].

In T cells, this expansion largely depends on the quality of the activation signal. Activation of functional T cells requires three signals: (i) TCR stimulation by an antigen-loaded MHC, (ii) co-stimulation involving interactions between molecules on the surface of APCs and T cells [22], and (iii) secretion of soluble cytokines crucial for signaling, proliferation, and differentiation of activated T cells into effector cells [23]. Signal ii can activate T cells when mediated by co-stimulatory molecules like CD28:CD80/86 or inhibit effector functions when mediated by co-inhibitory receptors such as CTLA-4:CD80/86; PD1:PDL1, TIM3, LAG3.

To demonstrate the stimulation of antigen specific CD8 + T cells, autologous DCs are often used as APCs because they express co-stimulatory molecules crucial for the survival and expansion of lymphocytes. However, their *in vitro* derivation is time-consuming, and their yields are not very high. To expand antigen specific CD8 + T cells against tumor antigens, some authors have proposed the creation of artificial APCs using cell lines that are easily co-transfectable with MHC-I haplotypes highly prevalent in the Caucasian population (e.g., HLA-A02:01, HLA-A24:02, and HLA-A * 03:01), plasmids encoding one or more co-stimulatory molecules, and minigenes encoding antigens of interest [24].

In this study, HEK293 cells were used as antigen presenting cells transfected with a minigene encoding epitopes and co-transfected with plasmids encoding co-stimulatory molecules. A comparison was conducted to evaluate which cells efficiently stimulate specific CD8 + T cells for viral and tumor antigens. To achieve this, the expression of intracellular cytokines and activation and exhaustion markers was assessed in CD8+ T cells present in PBMCs from healthy donors as well as in antigen specific CD8+ T cell clones. To confirm minigene delivery, GFP expression was monitored as a reporter gene.

To construct the artificial antigen-presenting cells (aAPCs), HEK293 cells were used and co-transfected with plasmids encoding CD80, CD83, CD137L, and the minigene. These cells demonstrated high transfection efficiency and expression of both co-stimulatory molecules and GFP (Fig 4). An important aspect to highlight is that transfecting the GFP-Minigene fusion protein consistently reduced the ability of GFP to emit fluorescence, which we attribute to the presence of the minigene that translates into a fusion protein with GFP (Fig 3). However, using GFP as a reporter gene fused with the minigene allowed us to monitor its transfection since its expression was not completely lost.

To verify the efficiency of aAPCs presentation, PBMCs from healthy donors were initially used, followed by antigen specific CD8 + T cell clones, which allowed controlling the variability related to the number of precursors present in the PBMCs. The use of HEK293 cells as aAPCs clearly demonstrated the production of intracellular cytokines by lymphocytes stimulated with these transfected aAPCs carrying the minigene and plasmids encoding co-stimulatory molecules. It was also evidenced that transfection with the minigene alone was able to stimulate an intracellular cytokine response equal to or greater than that presented with cells co-transfected with the co-stimulatory molecules and the minigene.

Within the evaluated intracellular cytokines was TNF-α (tumor necrosis factor alpha), which is a potent inflammatory cytokine capable of inducing a complex immune response and exerting antitumor effects by promoting apoptosis of cancer cells and destruction of tumor vasculature [25,26]. IFN-γ (interferon gamma) secretion was also assessed, which is a type II interferon that plays a crucial role in viral infections when viral mechanisms inhibit type I interferon function, in infection prevention, and in regulating inflammatory responses [27–29]. Within the populations of CD8+ T cells, polyfunctional lymphocytes expressing IFN-γ+ and TNF-α+ were observed. This is favorable for immunotherapy as they exhibit greater antitumor or antiviral efficacy by being able to perform multiple effector functions [29]. Additionally, populations of CD8+ T cells producing only IFN-γ+ were found to be dependent on the presence of HEK293 cells transfected with the minigene in the coculture, both in CD8 + T cells derived from PBMCs and from antigen specific clones.

Regarding populations of antigen-specific CD8+ T cells producing only TNF-α, a difference was observed between the response of CD8+ T cells expanded in PBMCs from healthy donors and in antigen-specific CD8 + T cell clones. While in PBMC-derived T cells there was non-specific expansion of the CD8 + TNF-α+ population when stimulated with HEK293 cells transfected only with GFP and in some cases in coculture with untransfected HEK293 cells, CMV + and Mart-1+ CD8+ T cell clones showed expansion of antigen-specific CD8 + T cells induced by aAPCs transfected with the minigene + /- co-stimulatory molecules, which was not observed when the clones were cocultured with untransfected HEK293 cells or cells transfected only with GFP. The fact that non-specific expansion of the CD8+ TNF-α+ antigen was detected only in CD8 + T cells present in blood from healthy donors and not in antigen-specific CD8+ T cell clones may be attributable to the presence of cellular populations present in PBMCs that induced TNF-α+ expression only in CD8 + T cells in response to HEK293 cells, which were not present in the coculture of HEK293 cells with pure CD8+ T cell clones.

Furthermore, in the populations of Mart-1 + CD8+ T cells stimulated by transfected HEK293 cells, in addition to intracellular cytokine expression, secretion of Granzyme B was evaluated. This protease functions to eliminate virus-infected cells and tumor cells [30]. The combined expression of intracellular cytokines and production of Granzyme B by purified CD8 + T cell clones confirm the utility of using clones as a tool to demonstrate the high immunogenicity of aAPCs based on HEK293 cells transfected with the minigene.

Among the molecules used for co-stimulation is CD80, which is the ligand for CD28 and is part of signal 2 for T cell activation. It signaling is vital for the development of T cell responses [31]. Another molecule used was CD83, mainly found on the membrane of mDCs. When encountering its ligand, it is associated with the stimulation of cytotoxic functions, regulation of maturation, activation of other immune cells, and homeostasis [32,33]. The last of the molecules used was CD137L, the ligand for CD137 (4-1BB). In CD8 + T cells, CD137/CD137L is known to increase proliferation, IFN-γ production, cytotoxic function, and survival [34].

The inclusion of co-stimulatory molecules was expected to enhance the stimulation of antigen specific CD8 + T cells. However, the addition of these molecules did not provide an advantage for the cytotoxic response of lymphocytes. This result may be explained by the fact that signal 2 activation of T cells, i.e., the interaction between CD28:CD80/86, initiates the activation and response process [35]. Considering that HEK293 cells express some CD80 (Fig 1), it is likely that increased CD80 expression does not necessarily translate into greater stimulation of CD8 + T cells; in other words, the signal from HEK293 cells may be sufficient to activate a response.

Hirano et al. (2006) mention that co-stimulatory molecules such as CD80/86, ICOS, 4-1BBL, and OX40L are induced after T cell activation and may take several days after T cell activation to reach their peak levels. This delay in their expression supports the hypothesis that they are not closely related to priming but rather with maintaining the response over time, supporting effector cell survival [33]. Considering that the coculture evaluated for intracellular cytokine production lasted only 6 hours, a longer follow-up may be required to demonstrate the impact of co-stimulation. However, it should be noted that a longer culture period would affect the optimal culture conditions for CD8 + T cells as HEK293 cells have a very high replication rate and can take control of the co-culture.

In addition to measuring intracellular cytokine expression, the expression of activation and exhaustion markers was evaluated. One of the markers studied was CD154 (also known as CD40L), which induces a potent signal for IL-2 production [36]. Therefore, the expression of this marker has been reported to play an important role in the expansion of CD8 + T cells in response to viral antigens [36].

It is well documented that the CD28:CD80/86 system is dependent on CD154 expression and upregulates markers such as CD25, CD137, and OX40 [37,38], which are activated T cell markers necessary for effector T cell function after stimulation and for the establishment of memory T cells [39]. In this work, through automated analysis tools, it was confirmed what some authors have suggested [40] that the use of co-stimulatory molecules in coculture favors CD154 expression in CD8 + T cells (Fig 8).

Another activation marker used was CD137 (4-1BB), which, when bound to CD137L, functions as a co-stimulatory signal, stimulating the proliferation of activated T cells, cytokine secretion, and anti-apoptotic signal [41]. These functions are mainly related to antiviral cytotoxic T cells [42]. Considering that its ligand was one of the co-stimulatory molecules used to transfect HEK293 cells, it makes sense that it was found in a higher proportion in CD8 + T cells present in PBMCs from healthy donors that were cocultured with aAPCs. However, since the expression of this marker is dependent on TCR activation, it also makes sense that it was found in CD8 + T cells stimulated with HEK293 cells transfected with the minigene [43].

CD25 was also used, which is considered one of the most important activation markers [44]. Its function is related to the activation of CD8 + T cells and IL-2 production [45]. It is known that it is expressed 24 hours after signal 1 and remains for several days [46]. Through automated analysis, it was found that the T cells did not express a large amount of CD25, possibly because the evaluation was conducted 24 hours after restimulation, and the peak expression of this marker may not have been reached.

CD69 was also evaluated as an early activation marker, and its function has been associated with a complex activating/regulating effect on the immune response. In this case, CD69 did not allow for the differentiation of antigen specific CD8 + T cells as expression was found in all cocultures. This may be due to the complex function of CD69, which involves regulating the response and its basal expression in inactivated lymphocytes. Additionally, its peak expression is reached rapidly, so a non-specific response may have been observed at 24 hours due to the presence of HEK293 cells and not antigenic stimulation.

Moving on to the exhaustion phenotype of CD8 + T cells, the expression of programmed cell death-1 (PD-1) was evaluated. This is an immunological checkpoint that downregulates T cell activity [47]. It has been shown that PD-1 is also expressed at early stages of activation in viral infections, which is related to the regulation of the T cell immune response [48]. Another exhaustion marker evaluated was cytotoxic T-lymphocyte-associated antigen 4 (CTLA-4), which is also considered an immunological checkpoint. This is a homolog of CD28, so it binds to CD80/86 but with an affinity 20 times higher than CD28 and inhibits cell proliferation by interrupting TCR signaling [49,50]. Similar to PD-1, CTLA-4 is also transiently expressed in in vitro activated CD8 + T cells because inducing a deficiency in this exhaustion marker leads to fatal lymphoproliferation in mice [51], demonstrating that its early expression is necessary to regulate the potency of the immune response [52].

The last exhaustion marker evaluated was lymphocyte-activation gene 3 (LAG-3 or CD223). Like the other two exhaustion markers, its function is to regulate the homeostasis of CD8 + T cell proliferation and activation [53]. Its ligand is MHC-II and considering that HEK293 cells express it on their surface, CD8 + T cells stimulated with these cells transfected with the minigene + /- co-stimulatory molecules showed an increase in this marker. Similar to the other two markers described previously, LAG-3 is expressed early after CD8 + T cell activation, as evidenced in mice transfused with LAG-3 knockout CD8 + T cells, which showed increased CD8 + T cell proliferation and cytokine production [54].

Exhaustion markers have traditionally been described as indicators of T cell dysfunction, representing cells that are exhausted and may not trigger an effective effector response. However, it has recently been shown that the expression of these markers may be associated with transcription factors involved in T cell activation and differentiation [55]. This explains why their presence was observed in a 24-hour coculture. It was observed that untransfected HEK293 cells stimulate a population of PD-1, which could be explained by the allogeneic nature of the coculture [56]. The expression of the other two markers was shown to be dependent on the presence of the antigen.

On the other hand, a comparison was made between the performance of mDCs stimulated with short peptides, long peptides, and recombinant protein of HLA-A * 0201 antigens from CMV and Mart-1 with aAPCs based on HEK293 cells co-transfected with the minigene and with plasmids encoding co-stimulatory molecules. It was found that aAPCs present a higher efficiency in presenting antigens processed naturally compared to the use of mDCs stimulated with recombinant protein from CMV or Mart-1. Additionally, mDCs stimulated with short or long peptides of these antigens did not significantly outperform HEK293 cells transfected. This suggests that the methodology proposed in this work, involving the modification of HEK293 cells to be used as artificial APCs, could be beneficial for identifying CD8 + T cell precursors for immunogenic neoantigens due to the efficiency of these cells in presenting antigens processed naturally.

In summary, this study presents an innovative platform based on HEK293 cells transfected with a minigene encoding for immunogenic epitopes restricted to the HLA-A * 0201 haplotype. The main purpose of this platform is to identify populations of antigen specific CD8 + T cells. It is interesting to note that transfection with plasmids encoding co-stimulatory molecules did not lead to a significant increase in intracellular cytokine expression, suggesting that antigen presentation is sufficient to stimulate a cytotoxic response. However, these molecules played an important role in stimulating the expression of activation and exhaustion markers in both CD8 + and CD4 + T cells.

This method has significant implications for identifying immunogenic neoantigens for cancer immunotherapy. The identification of CD8 + T cells using aAPCs transfected with minigenes in patient blood or tumor samples during the process of selecting tumor neoantigens is likely to lead to better selection of candidates for inclusion in personalized therapeutic vaccines using mDCs pulsed with neoantigens.

## Supporting information

S1 Fig Assessment of Markers Expressed by HEK293 Cells. The expression of HLA-DR and co-stimulatory molecules such as CD80 and CD83, along with CD14 and CD40 markers, was evaluated via flow cytometry using specific antibodies (Blue) compared to unmarked cells (Red).
(TIF)

S2 Fig HEK293 Cells Express HLA-A2. Representative histograms of HLA-A2 expression pattern in a) PBMCs and b) HEK293T cells labeled with the BB7 antibody (anti-HLA-A2) labeled with PE (red histogram) and unlabeled cells (blue).
(TIF)

S3 Fig Transfection using lipofectamine of HEK293T cells with pcDNA 3.1-N-eGFP plasmid.
Bars showing MFI levels of GFP in HEK293 cells transfected with pcDNA 3.1-N-eGFP-Minigene plasmid encoding GFP-Minigene fusion protein (GFP-Minigene), pcDNA 3.1-N-eGFP plasmid encoding GFP alone (GFP) and untransfected HEK293 cells. Bars represent mean +  SEM of 3 replicates, *  p-Value =  0.0046 (Kruskal-Wallis test).
(TIF)

S4 Fig Intracellular cytokine expression in CD8 + T cells from healthy donors cocultured with HEK293 cells as aAPCs.
Bars representing the fold change of populations of IFN-γ+ TNFα- CD8 + T cells from 4 healthy donors cocultured with HEK293 cells transfected with Molecules-GFP-Minigene, GFP-Minigene, GFP alone, untransfected cells, compared to the positive control (PBMCs stimulated with the CD8 epitope from CMV) and the negative control (unstimulated PBMCs). Statistical analysis of the groups was conducted using the non-parametric Kruskal-Wallis test, n = 4, (p < 0.05). The bars represent independent experimental replicates +  SEM.
(TIF)

S5 Fig Structure of the CITRUS analysis of CD8 T lymphocytes present in PBMCs from donor 042.
A) Expression tree of activation and exhaustion markers. B) Clustering tree indicating populations identified as statistically different by the program (red).
(TIF)

S6 Fig Results structure of the CITRUS analysis for CD8- T cells donor 042.
A) Box and whisker plot of the abundance of each CD3 CD8- population of PBMCs cultured with HEK293 cells transfected with Molecules-GFP-Minigene (blue) and PBMCs cultured with HEK293 cells transfected with GFP-Minigene (red). B) Expression histograms of markers in the populations identified in panel c (red), comparing background expression (blue).
(TIF)

S7 Fig Intracellular Cytokine Expression of CD8 + T Lymphocytes from Healthy Donors Co-cultured with Autologous or Artificial Antigen-Presenting Cells. Representative bar charts of the fold change or increase times of populations of CD8 + T lymphocytes IFN-γ+TNFα- (a) and IFN-γ-TNFα+ (b) from 3 healthy donors co-cultured with HEK293 cells transfected with Molecules-GFP-Minigene, GFP-Minigene, GFP only, untransfected, mDCs stimulated with short peptide, long peptide, and complete CMV protein, and unstimulated mDCs. PBMCs stimulated with the CD8 epitope of CMV were used as positive controls, and unstimulated PBMCs as negative controls. Statistical analysis of the groups was performed using the non-parametric Kruskal-Wallis test, n = 3, (p < 0.05). Bars represent independent experimental replicates +  SEM.
(TIF)

S8 Fig Product of PCR colony after transforming JM109 with the plasmid pcDNA.3.1-N-eGFP-minigene. 1.5% agarose gel of colony PCR products from plasmid pcDNA.3.1-N-eGFP-Minigene ligated in two different ratios (4:1 and 6:1 PCR product: recipient plasmid) with Bam-HI – XbaI digestion. A 50 bp DNA ladder was used, ranging from 2000 to 50 bp in size.
(TIF)

S9 Fig Plasmid pcDNA 3.1-N-EGFP-Minigen sequencing at Macrogen. 440 ng/ µ L of the plasmid obtained after ligating the minigene to the vector were sent to Macrogen for sequencing using the Sanger method (CES) in a standard sequencing type. The section containing the minigene sequence is highlighted in black.
(TIF)

S1 Table Selected CD8 Epitopes and Final Minigene Design with Furin Protease Spacer.
(XLSX)

S2 Table Healthy donors HLA-A * 0201 CMV + by double tetramer staining. (XLSX)

S3 Table Summary of fold change in IFN-γ+ TNF-α+ cells and IFN-γ+ TNF-α- cells for CMV and MART-1 in healthy donors and CD8 + T cell clones. (XLSX)

S1 File Supplementary Methods Minigene Design and Vector Selection.
(DOCX)

S2 File Raw data used for statistical analysis.
(XLSX)

## Abbreviations

APC: Antigen, Presenting, Cells
CFSE: Carboxyfluorescein, succinimidyl, ester
CTLA-4: Cytotoxic, T-Lymphocyte, Antigen, 4
DCs: Dendritic, Cells
DMSO: Dimethyl, sulfoxide
FITC: Fluorescein, IsoTioCyanate
GM-CSF: Granulocyte-macrophage, colony, stimulating, factor
HLA: Human, leukocyte, antigen
IFN: Interferon
LAG3: Lymphocyte-activation, gene, 3
IL: Interleukin
LPS: Lipopolysaccharide
LT-CD4 +: CD4 +, T-Lymphocyte
LT-CD8+: CD8+, T-Lymphocyte
MFI: Mean, fluorescence, intensity
MHC: Major, Histocompatibility, complex
MoDCs: Monocyte-derived, Dendritic, Cells
PB: Pacific, Blue
PBMCs: Peripheral, Blood, Mononuclear, Cells
PD-1: Programmed, cell, death, protein, 1
PE: Phycoerythrin
PRRs: Pattern, Recognition, Receptor
SFB: Fetal, Bovine, Serum
TILs: Tumor, Infiltrating, Lymphocytes
TLR: Toll-like, receptor
TNF: Tumor, necrosis, factor.

## References

[1] Tan S, Li D, Zhu X. Cancer immunotherapy: Pros, cons and beyond. Biomed Pharmacother. 2020;124:109821. 31962285 10.1016/j.biopha.2020.109821

[2] Dagher OK, Schwab RD, Brookens SK, Posey AD Jr. Advances in cancer immunotherapies. Cell. 2023;186(8):1814–e1. doi: 10.1016/j.cell.2023.02.039 37059073

[3] Zhang Y, Zhang Z. The history and advances in cancer immunotherapy: understanding the characteristics of tumor-infiltrating immune cells and their therapeutic implications. Cell Mol Immunol. 2020;17(8):807–21. doi: 10.1038/s41423-020-0488-6 32612154 PMC7395159

[4] Lollini P-L, Cavallo F, Nanni P, Forni G. Vaccines for tumour prevention. Nat Rev Cancer. 2006;6(3):204–16. doi: 10.1038/nrc1815 16498443

[5] Fu C, Zhou L, Mi Q-S, Jiang A. DC-Based Vaccines for Cancer Immunotherapy. Vaccines (Basel). 2020;8(4):706. doi: 10.3390/vaccines8040706 33255895 PMC7712957

[6] Devi GR, Nath S. Delivery of Synthetic mRNA Encoding FOXP3 Antigen into Dendritic Cells for Inflammatory Breast Cancer Immunotherapy. Methods Mol Biol. 2016;1428:231–43. doi: 10.1007/978-1-4939-3625-0_15 27236803

[7] Sahin U, Derhovanessian E, Miller M, Kloke B-P, Simon P, Löwer M, et al. Personalized RNA mutanome vaccines mobilize poly-specific therapeutic immunity against cancer. Nature. 2017;547(7662):222–6. doi: 10.1038/nature23003 28678784

[8] Carreno BM, Magrini V, Becker-Hapak M, Kaabinejadian S, Hundal J, Petti AA, et al. Cancer immunotherapy. A dendritic cell vaccine increases the breadth and diversity of melanoma neoantigen-specific T cells. Science. 2015;348(6236):803–8. doi: 10.1126/science.aaa3828 25837513 PMC4549796

[9] Patente TA, Pinho MP, Oliveira AA, Evangelista GCM, Bergami-Santos PC, Barbuto JAM. Human Dendritic Cells: Their Heterogeneity and Clinical Application Potential in Cancer Immunotherapy. Front Immunol. 2019;9:3176. doi: 10.3389/fimmu.2018.03176 30719026 PMC6348254

[10] Lesterhuis WJ, De Vries IJM, Schreibelt G, Schuurhuis DH, Aarntzen EH, De Boer A, et al. Immunogenicity of dendritic cells pulsed with CEA peptide or transfected with CEA mRNA for vaccination of colorectal cancer patients. Anticancer Res. 2010;30(12):5091–7. 21187495

[11] Wells DK, van Buuren MM, Dang KK, Hubbard-Lucey VM, Sheehan KCF, Campbell KM, et al. Key Parameters of Tumor Epitope Immunogenicity Revealed Through a Consortium Approach Improve Neoantigen Prediction. Cell. 2020;183(3):818–34.e13. doi: 10.1016/j.cell.2020.09.015 33038342 PMC7652061

[12] Cafri G, Gartner JJ, Zaks T, Hopson K, Levin N, Paria BC, et al. mRNA vaccine-induced neoantigen-specific T cell immunity in patients with gastrointestinal cancer. J Clin Invest. 2020;130(11):5976–88. doi: 10.1172/JCI134915 33016924 PMC7598064

[13] Aurisicchio L, Fridman A, Bagchi A, Scarselli E, La Monica N, Ciliberto G. A novel minigene scaffold for therapeutic cancer vaccines. Oncoimmunology. 2014;3(1):e27529. doi: 10.4161/onci.27529 24790791 PMC4002591

[14] Tateshita N, Miura N, Tanaka H, Masuda T, Ohtsuki S, Tange K, et al. Development of a lipoplex-type mRNA carrier composed of an ionizable lipid with a vitamin E scaffold and the KALA peptide for use as an ex vivo dendritic cell-based cancer vaccine. Journal of Controlled Release: Official Journal of the Controlled Release Society. 2019;310. 10.1016/j.jconrel.2019.08.002 31386869

[15] Lu Y-C, Yao X, Crystal JS, Li YF, El-Gamil M, Gross C, et al. Efficient identification of mutated cancer antigens recognized by T cells associated with durable tumor regressions. Clin Cancer Res. 2014;20(13):3401–10. doi: 10.1158/1078-0432.CCR-14-0433 24987109 PMC4083471

[16] Ménager J, Ebstein F, Oger R, Hulin P, Nedellec S, Duverger E, et al. Cross-presentation of synthetic long peptides by human dendritic cells: a process dependent on ERAD component p97/VCP but Not sec61 and/or Derlin-1. PLoS One. 2014;9(2):e89897. doi: 10.1371/journal.pone.0089897 24587108 PMC3937416

[17] Aspord C, Leloup C, Reche S, Plumas J. pDCs efficiently process synthetic long peptides to induce functional virus- and tumour-specific T-cell responses. Eur J Immunol. 2014;44(10):2880–92. doi: 10.1002/eji.201444588 25043392

[18] Foged C, Arigita C, Sundblad A, Jiskoot W, Storm G, Frokjaer S. Interaction of dendritic cells with antigen-containing liposomes: effect of bilayer composition. Vaccine. 2004;22(15–16):1903–13. doi: 10.1016/j.vaccine.2003.11.008 15121302

[19] Arnaud M, Chiffelle J, Genolet R, Navarro Rodrigo B, Perez MAS, Huber F, et al. Sensitive identification of neoantigens and cognate TCRs in human solid tumors. Nat Biotechnol. 2022;40(5):656–60. doi: 10.1038/s41587-021-01072-6 34782741 PMC9110298

[20] Liu Y, Li C, Lu Y, Liu C, Yang W. Tumor microenvironment-mediated immune tolerance in development and treatment of gastric cancer. Front Immunol. 2022;13:1016817. doi: 10.3389/fimmu.2022.1016817 36341377 PMC9630479

[21] Phetsouphanh C, Zaunders JJ, Kelleher AD. Detecting Antigen-Specific T Cell Responses: From Bulk Populations to Single Cells. Int J Mol Sci. 2015;16(8):18878–93. doi: 10.3390/ijms160818878 26274954 PMC4581277

[22] Azuma M. Co-signal Molecules in T-Cell Activation : Historical Overview and Perspective. Adv Exp Med Biol. 2019;1189:3–23. doi: 10.1007/978-981-32-9717-3_1 31758529

[23] Curtsinger JM, Schmidt CS, Mondino A, Lins DC, Kedl RM, Jenkins MK, et al. Inflammatory cytokines provide a third signal for activation of naive CD4+ and CD8+ T cells. J Immunol. 1999;162(6):3256–62. doi: 10.4049/jimmunol.162.6.3256 10092777

[24] Tanimoto K, Muranski P, Miner S, Fujiwara H, Kajigaya S, Keyvanfar K, et al. Genetically engineered fixed K562 cells: potent “off-the-shelf” antigen-presenting cells for generating virus-specific T cells. Cytotherapy. 2014;16(1):135–46. doi: 10.1016/j.jcyt.2013.08.008 24176543 PMC6755681

[25] Mortara L, Balza E, Sassi F, Castellani P, Carnemolla B, De Lerma Barbaro A, et al. Therapy-induced antitumor vaccination by targeting tumor necrosis factor alpha to tumor vessels in combination with melphalan. Eur J Immunol. 2007;37(12):3381–92. doi: 10.1002/eji.200737450 18022863

[26] Lejeune F, Liénard D, Matter M, Rüegg C. Efficiency of recombinant human TNF in human cancer therapy. Cancer Immunology. 2006;6(6). 16551058

[27] Kang S, Brown H, Hwang S. Direct antiviral mechanisms of interferon-gamma. Immune Network. 2018;18(5):e33. 30402328 10.4110/in.2018.18.e33 PMC6215902

[28] Jouanguy E, Lamhamedi-Cherradi S, Lammas D, Dorman S, Fondanèche M, Dupuis S. A human IFNGR1 small deletion hotspot associated with dominant susceptibility to mycobacterial infection. Nature Genetics. 1999;21(4):370–8. 10192386 10.1038/7701

[29] Ikeda H, Old LJ, Schreiber RD. The roles of IFN gamma in protection against tumor development and cancer immunoediting. Cytokine Growth Factor Rev. 2002;13(2):95–109. doi: 10.1016/s1359-6101(01)00038-7 11900986

[30] Trapani J, Sutton V. Granzyme B: pro-apoptotic, antiviral and antitumor functions. Current Opinion in Immunology. 2003;15(5):533–43. 14499262 10.1016/s0952-7915(03)00107-9

[31] Melichar B, Nash MA, Lenzi R, Platsoucas CD, Freedman RS. Expression of costimulatory molecules CD80 and CD86 and their receptors CD28, CTLA-4 on malignant ascites CD3+ tumour-infiltrating lymphocytes (TIL) from patients with ovarian and other types of peritoneal carcinomatosis. Clin Exp Immunol. 2000;119(1):19–27. doi: 10.1046/j.1365-2249.2000.01105.x 10606960 PMC1905534

[32] Li Z, Ju X, Silveira PA, Abadir E, Hsu W-H, Hart DNJ, et al. CD83: Activation Marker for Antigen Presenting Cells and Its Therapeutic Potential. Front Immunol. 2019;10:1312. doi: 10.3389/fimmu.2019.01312 31231400 PMC6568190

[33] Hirano N, Butler MO, Xia Z, Ansén S, von Bergwelt-Baildon MS, Neuberg D, et al. Engagement of CD83 ligand induces prolonged expansion of CD8+ T cells and preferential enrichment for antigen specificity. Blood. 2006;107(4):1528–36. doi: 10.1182/blood-2005-05-2073 16239433 PMC1895397

[34] Shuford WW, Klussman K, Tritchler DD, Loo DT, Chalupny J, Siadak AW, et al. 4-1BB costimulatory signals preferentially induce CD8+ T cell proliferation and lead to the amplification in vivo of cytotoxic T cell responses. J Exp Med. 1997;186(1):47–55. doi: 10.1084/jem.186.1.47 9206996 PMC2198949

[35] de Jong R, Loenen WA, Brouwer M, van Emmerik L, de Vries EF, Borst J, et al. Regulation of expression of CD27, a T cell-specific member of a novel family of membrane receptors. J Immunol. 1991;146(8):2488–94. doi: 10.4049/jimmunol.146.8.2488 1707907

[36] Hassan GS, Stagg J, Mourad W. Role of CD154 in cancer pathogenesis and immunotherapy. Cancer Treat Rev. 2015;41(5):431–40. doi: 10.1016/j.ctrv.2015.03.007 25843228

[37] Sharpe A, Freeman G. The B7-CD28 superfamily. Nature Reviews Immunol. 2002;2(2):116–26. 10.1038/nri727 11910893

[38] Walker LS, Gulbranson-Judge A, Flynn S, Brocker T, Lane PJ. Co-stimulation and selection for T-cell help for germinal centres: the role of CD28 and OX40. Immunol Today. 2000;21(7):333–7. doi: 10.1016/s0167-5699(00)01636-4 10871874

[39] Acuto O, Michel F. CD28-mediated co-stimulation: a quantitative support for TCR signalling. Nat Rev Immunol. 2003;3(12):939–51. doi: 10.1038/nri1248 14647476

[40] Kaminski DA, Lee BO, Eaton SM, Haynes L, Randall TD. CD28 and inducible costimulator (ICOS) signalling can sustain CD154 expression on activated T cells. Immunology. 2009;127(3):373–85. doi: 10.1111/j.1365-2567.2008.02991.x 19191918 PMC2712106

[41] Dawicki W, Watts TH. Expression and function of 4-1BB during CD4 versus CD8 T cell responses in vivo. Eur J Immunol. 2004;34(3):743–51. doi: 10.1002/eji.200324278 14991604

[42] Otano I, Azpilikueta A, Glez-Vaz J, Alvarez M, Medina-Echeverz J, Cortés-Domínguez I, et al. CD137 (4-1BB) costimulation of CD8+ T cells is more potent when provided in cis than in trans with respect to CD3-TCR stimulation. Nat Commun. 2021;12(1):7296. doi: 10.1038/s41467-021-27613-w 34911975 PMC8674279

[43] Wolfl M, Kuball J, Ho W, Nguyen H, Manley T, Bleakley M. Activation-induced expression of CD137 permits detection, isolation, and expansion of the full repertoire of CD8+ T cells responding to antigen without requiring knowledge of epitope specificities. Blood. 2007;110(1):201–10. 17371945 10.1182/blood-2006-11-056168 PMC1896114

[44] Bajnok A, Ivanova M, Rigó J Jr, Toldi G. The Distribution of Activation Markers and Selectins on Peripheral T Lymphocytes in Preeclampsia. Mediators Inflamm. 2017;2017:8045161. doi: 10.1155/2017/8045161 28555090 PMC5438859

[45] Rea I, McNerlan S, Alexander H. CD69, CD25, and HLA-DR activation antigen expression on CD3+ lymphocytes and relationship to serum TNF-alpha, IFN-gamma, and sIL-2R levels in aging. Exp Gerontol. 1999;34(1):79–93. 10197730 10.1016/s0531-5565(98)00058-8

[46] Reddy M, Eirikis E, Davis C, Davis HM, Prabhakar U. Comparative analysis of lymphocyte activation marker expression and cytokine secretion profile in stimulated human peripheral blood mononuclear cell cultures: an in vitro model to monitor cellular immune function. J Immunol Methods. 2004;293(1–2):127–42. doi: 10.1016/j.jim.2004.07.006 15541283

[47] Jubel JM, Barbati ZR, Burger C, Wirtz DC, Schildberg FA. The Role of PD-1 in Acute and Chronic Infection. Front Immunol. 2020;11:487. doi: 10.3389/fimmu.2020.00487 32265932 PMC7105608

[48] Ahn E, Araki K, Hashimoto M, Li W, Riley JL, Cheung J, et al. Role of PD-1 during effector CD8 T cell differentiation. Proc Natl Acad Sci U S A. 2018;115(18):4749–54. doi: 10.1073/pnas.1718217115 29654146 PMC5939075

[49] Pentcheva-Hoang T, Egen JG, Wojnoonski K, Allison JP. B7-1 and B7-2 selectively recruit CTLA-4 and CD28 to the immunological synapse. Immunity. 2004;21(3):401–13. doi: 10.1016/j.immuni.2004.06.017 15357951

[50] Walunas TL, Sperling AI, Khattri R, Thompson CB, Bluestone JA. CD28 expression is not essential for positive and negative selection of thymocytes or peripheral T cell tolerance. J Immunol. 1996;156(3):1006–13. 8557973

[51] Waterhouse P, Penninger JM, Timms E, Wakeham A, Shahinian A, Lee KP, et al. Lymphoproliferative disorders with early lethality in mice deficient in Ctla-4. Science. 1995;270(5238):985–8. doi: 10.1126/science.270.5238.985 7481803

[52] Steiner K, Waase I, Rau T, Dietrich M, Fleischer B, Bröker BM. Enhanced expression of CTLA-4 (CD152) on CD4+ T cells in HIV infection. Clin Exp Immunol. 1999;115(3):451–7. doi: 10.1046/j.1365-2249.1999.00806.x 10193417 PMC1905266

[53] Workman CJ, Cauley LS, Kim IJ, Blackman MA, Woodland DL, Vignali DA. Lymphocyte activation gene-3 (CD223) regulates the size of the expanding T cell population following antigen activation in vivo. J Immunol. 2004;172(9):5450–5. 15100286 10.4049/jimmunol.172.9.5450

[54] Goldberg MV, Drake CG. LAG-3 in Cancer Immunotherapy. Curr Top Microbiol Immunol. 2011;344:269–78. doi: 10.1007/82_2010_114 21086108 PMC4696019

[55] Fuertes Marraco SA, Neubert NJ, Verdeil G, Speiser DE. Inhibitory Receptors Beyond T Cell Exhaustion. Front Immunol. 2015;6:310. doi: 10.3389/fimmu.2015.00310 26167163 PMC4481276

[56] Wang W, Carper K, Malone F, Latchman Y, Perkins J, Fu Y, et al. PD-L1/PD-1 signal deficiency promotes allogeneic immune responses and accelerates heart allograft rejection. Transplantation. 2008;86(6):836–44. doi: 10.1097/TP.0b013e3181861932 18813109

