# Supplementary figures and images for: Assessment of antigen immunogenicity formulated in minigenes transfected into antigen-presenting cells
Source: PLoS One. 2025 Apr 7;20(4):e0321392. doi: 10.1371/journal.pone.0321392 (PMC11975385; doi:10.1371/journal.pone.0321392)

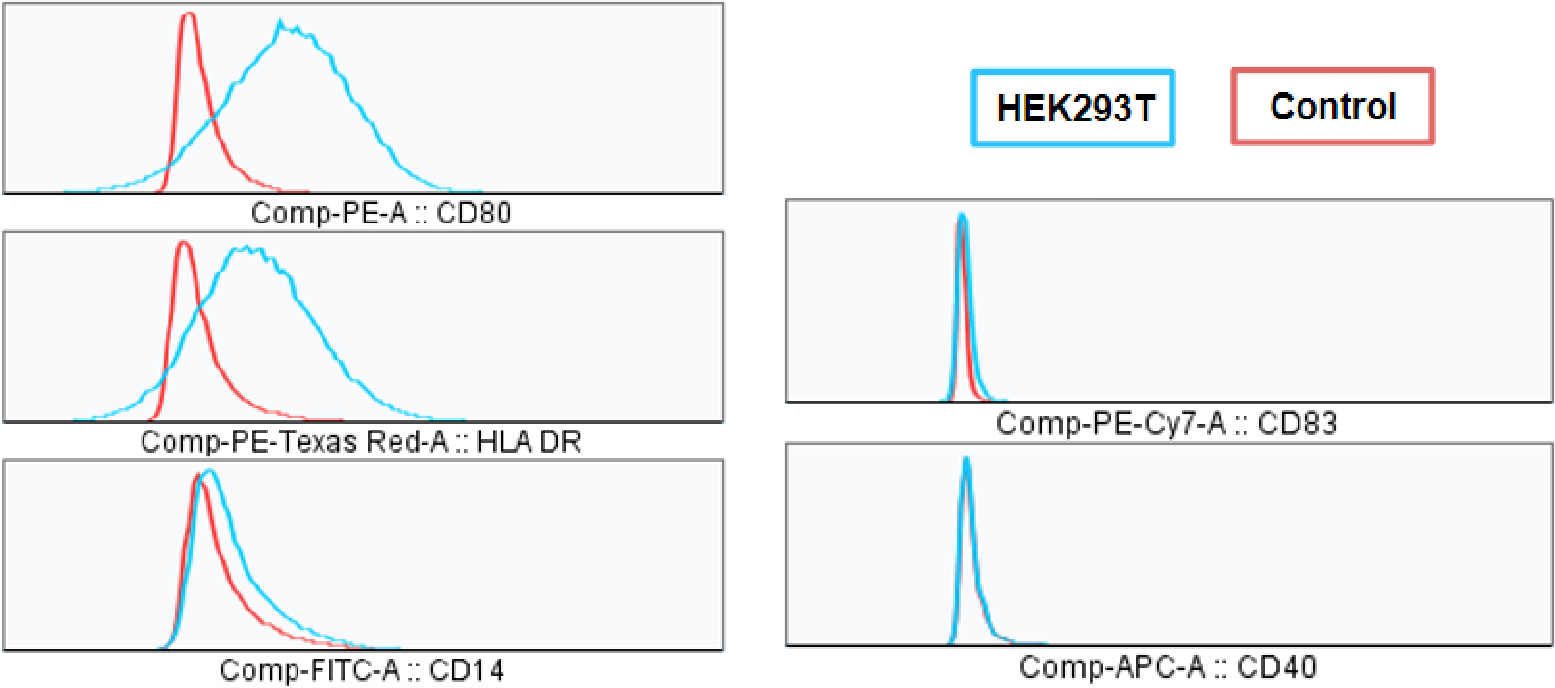

Supplement: S1 Fig — The expression of HLA-DR and co-stimulatory molecules such as CD80 and CD83, along with CD14 and CD40 markers, was evaluated via flow cytometry using specific antibodies (Blue) compared to unmarked cells (Red). (TIF) [file pone.0321392.s001.tif]

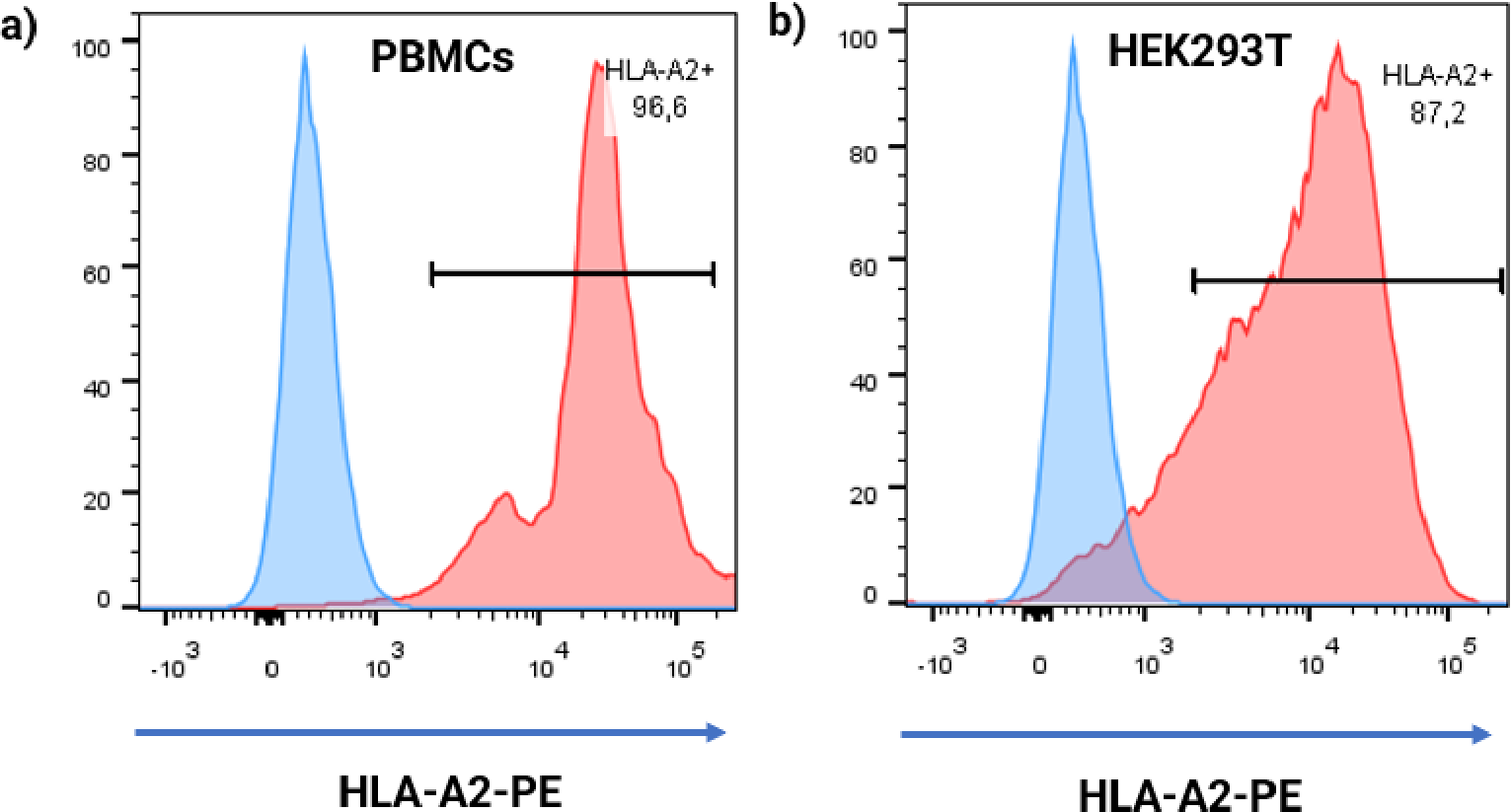

Supplement: S2 Fig — Representative histograms of HLA-A2 expression pattern in a) PBMCs and b) HEK293T cells labeled with the BB7 antibody (anti-HLA-A2) labeled with PE (red histogram) and unlabeled cells (blue). (TIF) [file pone.0321392.s002.tif]

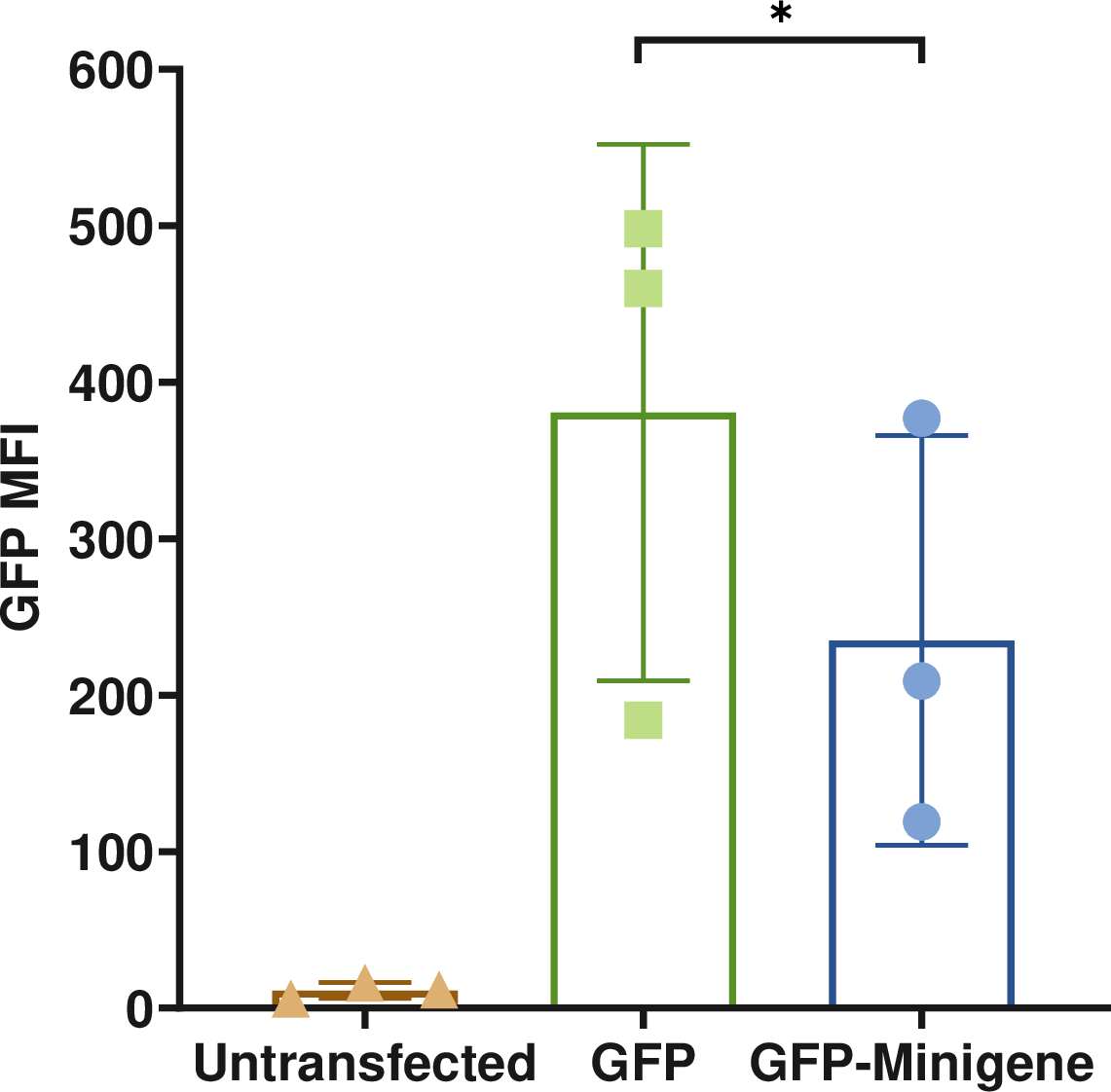

Supplement: S3 Fig — Bars showing MFI levels of GFP in HEK293 cells transfected with pcDNA 3.1-N-eGFP-Minigene plasmid encoding GFP-Minigene fusion protein (GFP-Minigene), pcDNA 3.1-N-eGFP plasmid encoding GFP alone (GFP) and untransfected HEK293 cells. Bars represent mean + SEM of 3 replicates, * p-Value = 0.0046 (Kruskal-Wallis test). (TIF) [file pone.0321392.s003.tif]

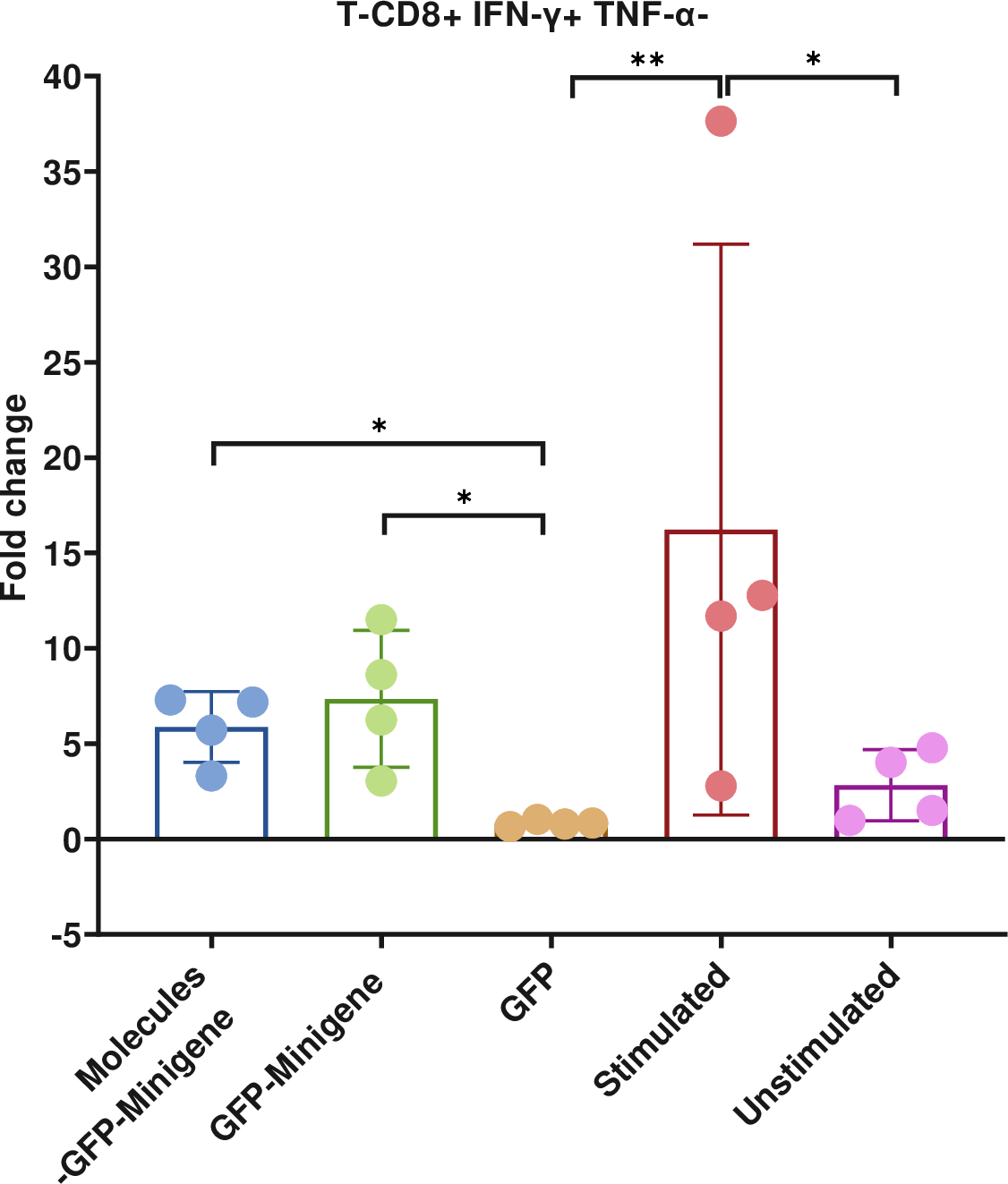

Supplement: S4 Fig — Bars representing the fold change of populations of IFN-γ+ TNFα- CD8 + T cells from 4 healthy donors cocultured with HEK293 cells transfected with Molecules-GFP-Minigene, GFP-Minigene, GFP alone, untransfected cells, compared to the positive control (PBMCs stimulated with the CD8 epitope from CMV) and the negative control (unstimulated PBMCs). Statistical analysis of the groups was conducted using the non-parametric Kruskal-Wallis test, n = 4, (p < 0.05). The bars represent independent experimental replicates + SEM. (TIF) [file pone.0321392.s004.tif]

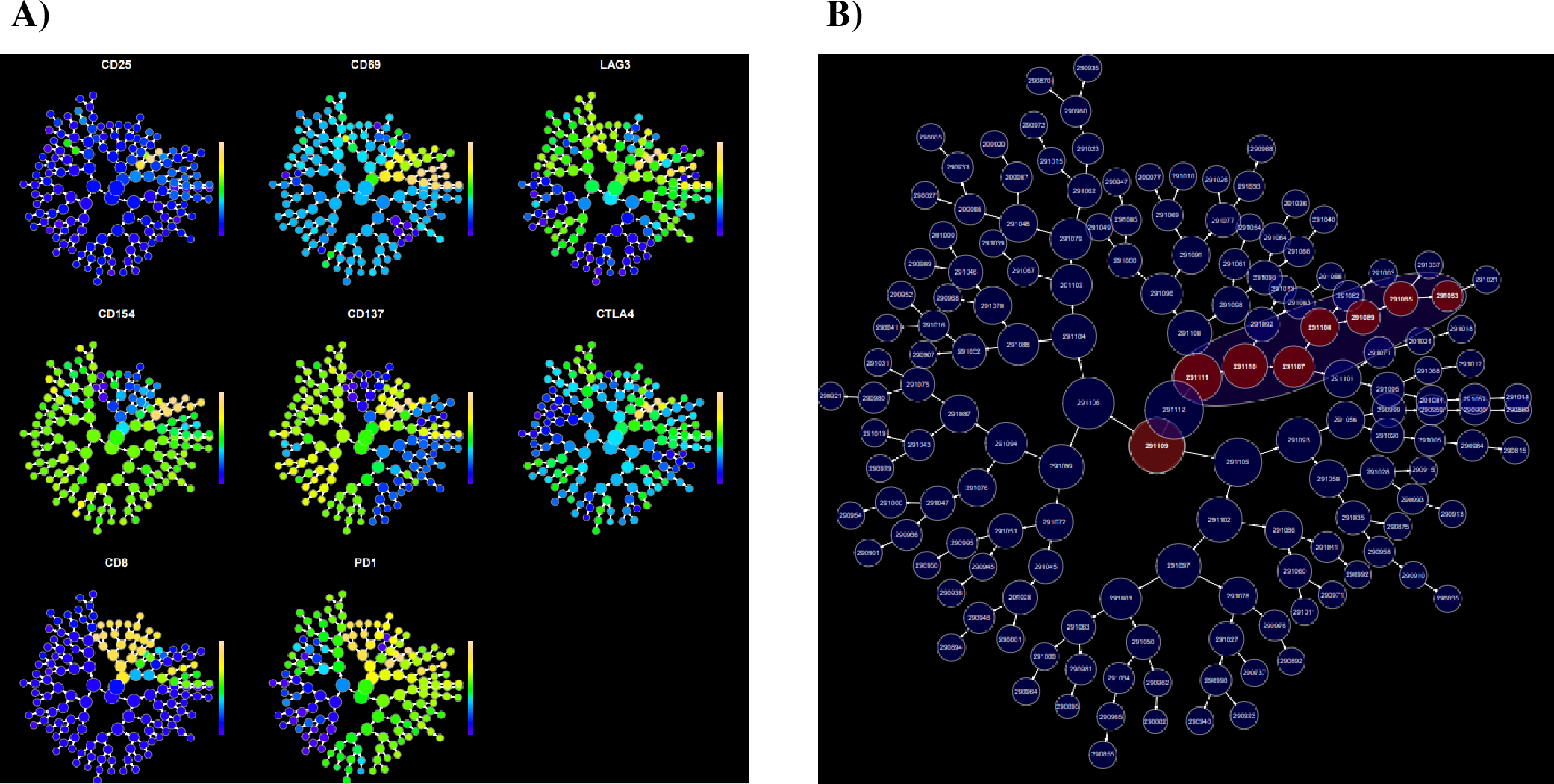

Supplement: S5 Fig — A) Expression tree of activation and exhaustion markers. B) Clustering tree indicating populations identified as statistically different by the program (red). (TIF) [file pone.0321392.s005.tif]

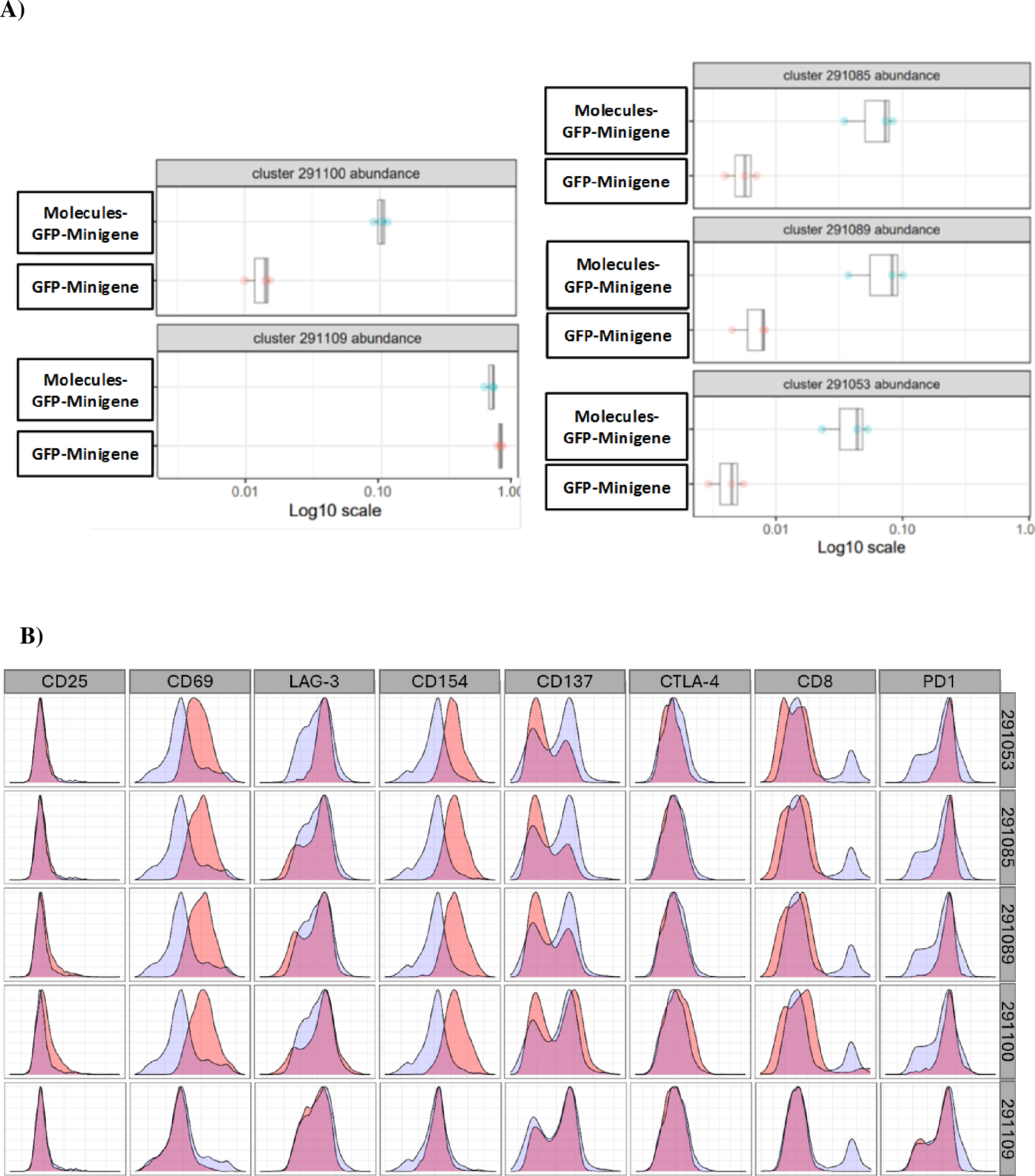

Supplement: S6 Fig — A) Box and whisker plot of the abundance of each CD3 CD8- population of PBMCs cultured with HEK293 cells transfected with Molecules-GFP-Minigene (blue) and PBMCs cultured with HEK293 cells transfected with GFP-Minigene (red). B) Expression histograms of markers in the populations identified in panel c (red), comparing background expression (blue). (TIF) [file pone.0321392.s006.tif]

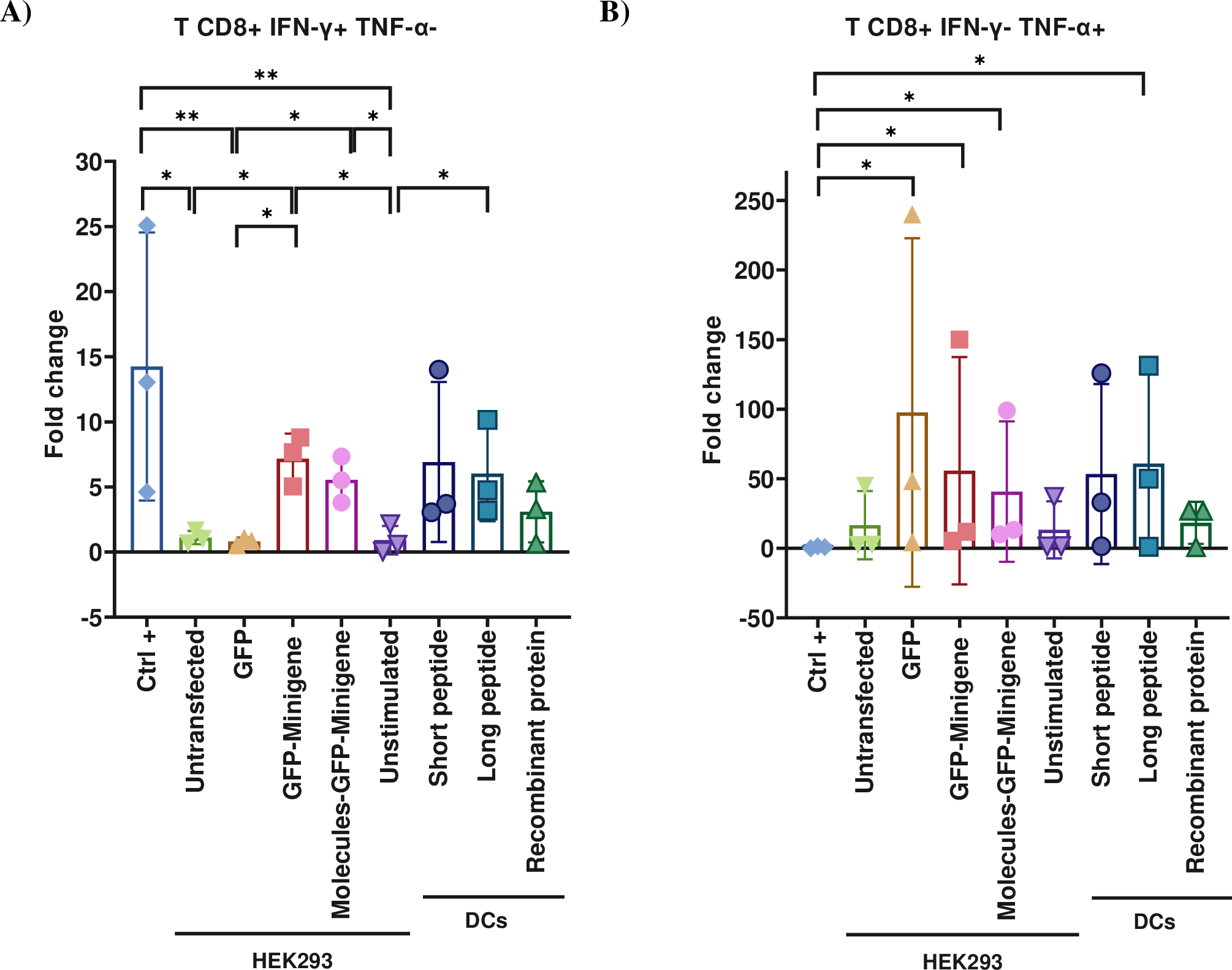

Supplement: S7 Fig — Representative bar charts of the fold change or increase times of populations of CD8 + T lymphocytes IFN-γ+TNFα- (a) and IFN-γ-TNFα+ (b) from 3 healthy donors co-cultured with HEK293 cells transfected with Molecules-GFP-Minigene, GFP-Minigene, GFP only, untransfected, mDCs stimulated with short peptide, long peptide, and complete CMV protein, and unstimulated mDCs. PBMCs stimulated with the CD8 epitope of CMV were used as positive controls, and unstimulated PBMCs as negative controls. Statistical analysis of the groups was performed using the non-parametric Kruskal-Wallis test, n = 3, (p < 0.05). Bars represent independent experimental replicates + SEM. (TIF) [file pone.0321392.s007.tif]

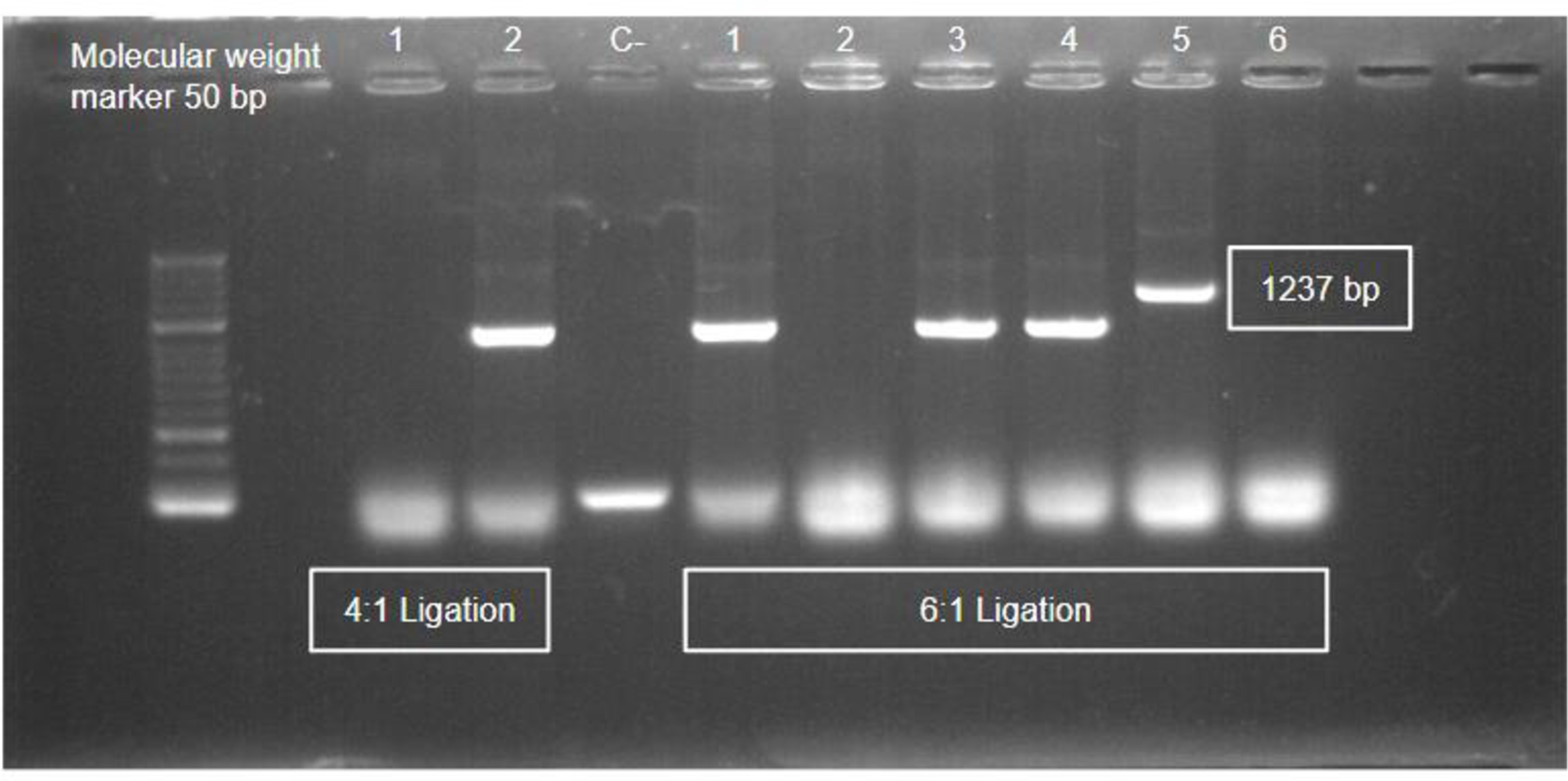

Supplement: S8 Fig — 1.5% agarose gel of colony PCR products from plasmid pcDNA.3.1-N-eGFP-Minigene ligated in two different ratios (4:1 and 6:1 PCR product: recipient plasmid) with Bam-HI – XbaI digestion. A 50 bp DNA ladder was used, ranging from 2000 to 50 bp in size. (TIF) [file pone.0321392.s008.tif]

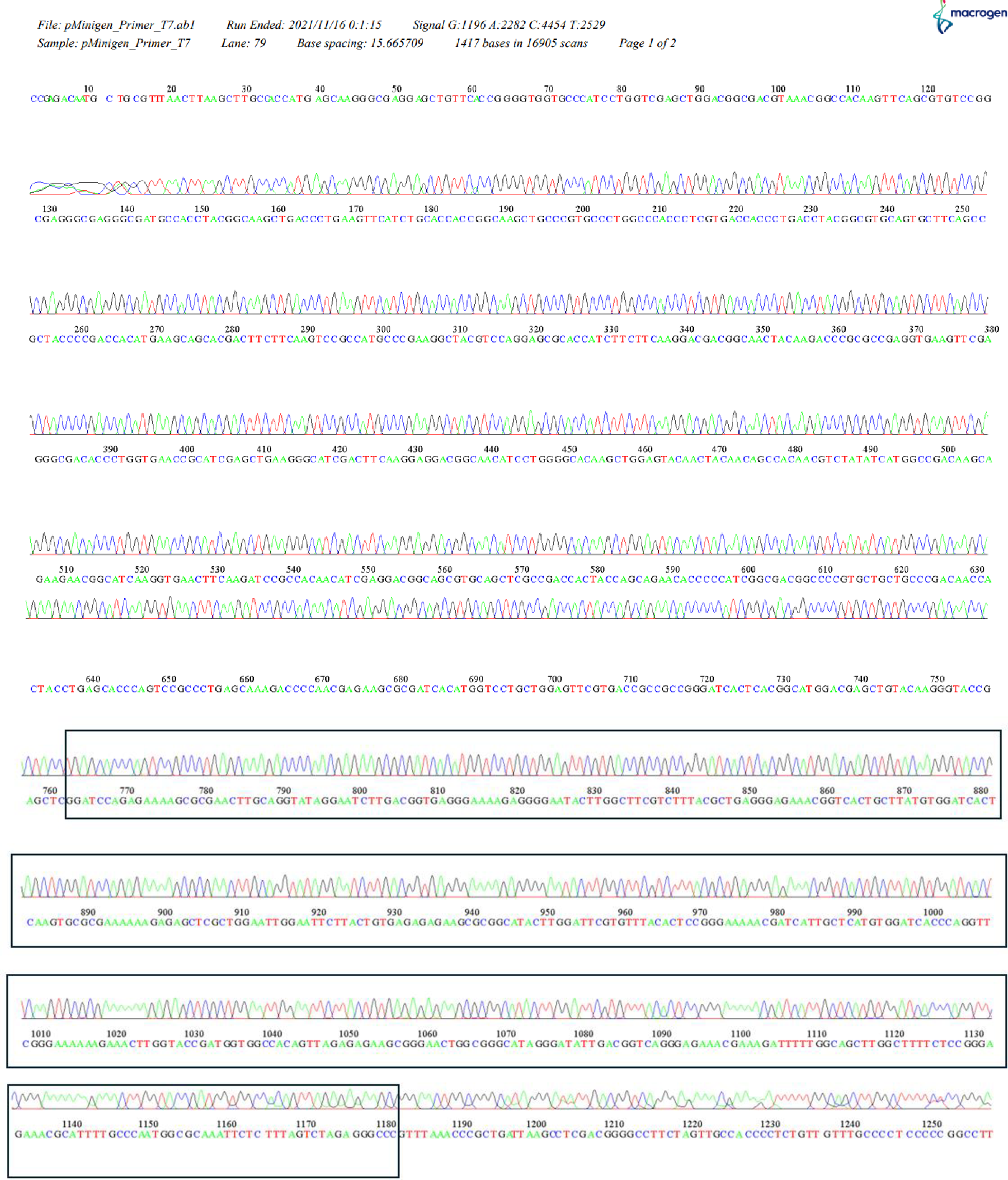

Supplement: S9 Fig — 440 ng/ µ L of the plasmid obtained after ligating the minigene to the vector were sent to Macrogen for sequencing using the Sanger method (CES) in a standard sequencing type. The section containing the minigene sequence is highlighted in black. (TIF) [file pone.0321392.s009.tif]
