# Supplementary material for: Assessment of antigen immunogenicity formulated in minigenes transfected into antigen-presenting cells
Source: PLoS One. 2025 Apr 7;20(4):e0321392. doi: 10.1371/journal.pone.0321392 (PMC11975385; doi:10.1371/journal.pone.0321392)
Supplement: S1 File — (DOCX) [file pone.0321392.s013.docx]

**Supplementary Materials**

**Minigene Design and Vector Selection**

In line with the methodology described, the minigene was designed to encode multiple epitopes restricted to the HLA-A*02:01 allele, whose immunogenicity for human CD8+ T cells has been extensively demonstrated. Table S1 outlines the amino acid sequences of the selected epitopes, each separated by the furin protease recognition sequence, along with the position of each antigen within the minigene. The final minigene product was synthesized within the pUC-IDT plasmid (Integrated DNA Technologies IDT), wherein the sequences of each epitope are repeated at least once. Before synthesis, the construct underwent analysis using the ProP.1.0 program from the University of Denmark to identify the furin protease cleavage pattern. Table S1 highlights the 10 cleavage sites, which precisely correspond to the locations of the spacers.

Furthermore, the Addgene’s Sequence Analyzer program was employed to identify the restriction enzyme cutting sites incorporated into the minigene, ensuring that the selected cloning enzymes were absent in the minigene. This analysis confirmed the presence of only one cutting site for the selected cloning enzymes.

The pcDNA 3.1-N-eGFP vector (GenScript) was chosen for its capability of minigene cloning and replication within eukaryotic cells. For minigene cloning, the BamHI enzyme was chosen for the 5' end, while the Xba-I enzyme was selected for the 3' end. To engineer these restriction enzyme sites, a polymerase chain reaction (PCR) was conducted using the PUC-IDT plasmid to insert the recognition sequences of the Bam-HI - Xba-I restriction enzyme pairs for minigene cloning into the pcDNA 3.1-N-eGFP vector.

Subsequently, to insert the minigene sequence into the pcDNA 3.1-N-eGFP plasmid, digestion of the PCR product and plasmid was carried out. Ligation was performed using T4 ligase enzyme (Invitrogen), incubating the linearized vector with the PCR product digested with Bam-HI and Xba-I enzymes in two different ratios: 4:1 and 6:1 (PCR product:plasmid receptor), along with the enzyme, for 15 minutes at room temperature. For the amplification of the resulting plasmid, competent JM109 cells were transformed with 5 µL of the selected ligation product. After 24 hours of bacterial culture, colonies that grew visibly without satellite colonies were harvested. For the PCR of recombinant colonies, only two colonies were used in the case of the 4:1 ratio, while six colonies were found from cultures with the 6:1 ratio. As seen in Figure S8, none of the colonies that grew with the first insert: vector ratio yielded the expected band. In contrast, one of the six colonies resulting from the transformation with the 6:1 ratio exhibited the band with the expected weight (1237 bp) for the final vector-minigene construct. For this reason, this colony was selected as the basis for the amplification and purification of the recombinant plasmid containing the minigene. Lastly, confirming the presence of the minigene in the plasmid sequencing of this plasmid was also performed, providing confirmation that the minigene sequence was as expected and that it was in the expected reading frame (Figure S9).
